# Association and Interaction Analysis of Body Mass Index and Triglycerides Level with Blood Pressure in Elderly Individuals in China

**DOI:** 10.1155/2018/8934534

**Published:** 2018-11-22

**Authors:** Lin Zhang, Jin-long Li, Li-li Zhang, Lei-lei Guo, Hong Li, Dan Li

**Affiliations:** ^1^Department of Community Nursing, School of Nursing, Jinzhou Medical University, No. 40, Section 3, Songpo Road, Linghe District Jinzhou City, Liaoning Province, China; ^2^Department of Occupational and Environmental Health, Key Laboratory of Occupational Health and Safety for Coal Industry in Hebei Province, School of Public Health, North China University of Science and Technology, Tangshan, Hebei Province, China; ^3^Department of Surgery, Third Affiliated Hospital of Jinzhou Medical University, No. 28, Section 2, Chongqing Road, Linghe District, Jinzhou City, Liaoning Province, China; ^4^Experimental Center for Nursing, School of Nursing, Jinzhou Medical University, No. 40, Section 3, Songpo Road, Linghe District Jinzhou City, Liaoning Province, China

## Abstract

**Objectives:**

To assess the extent of interaction between body mass index (BMI) and triglyceride (TG) level and its effects on blood pressure (BP) in elderly individuals in China.

**Design:**

Cross-sectional study.

**Setting:**

Data were taken from a cross-sectional study called the China Health and Retirement Longitudinal Study.

**Participants:**

The analytic sample included 3629 subjects aged 45 to 96 years.

**Main Outcome Measurements:**

Data were obtained from the China Health and Retirement Longitudinal Study, which is a cross-sectional study. Age-adjusted partial Pearson's correlation test was used to compare various characteristics and BP. Adjusted associations were first used as linear regression models, as appropriate. Then, general linear models adjusted for related potential confounders were used to examine the synergistic effects of BMI and TG level on BP. Finally, a binary logistic regression model adjusted for confounding factors was used to examine the association between BMI or TG level and hypertension.

**Results:**

Age-adjusted partial Pearson's correlation coefficient showed that the TG level was positively correlated with both systolic blood pressure (SBP) and diastolic blood pressure (DBP) in both men and women with BMI < 24.0 kg/m^2^; however, TG level was positively correlated with DBP in women with BMI ≥ 24.0 kg/m^2^ but not with DBP in men with BMI ≥ 24.0 kg/m^2^. Multiple linear regression analysis showed that BMI level was significantly and positively associated with both SBP and DBP in men and women with BMI < 24.0 kg/m^2^, and TG level was significantly and positively associated with SBP in women with BMI < 24.0 kg/m^2^, independent of other confounding factors. A general linear model analysis with adjustment for confounding factors (age, educational level, marital status, current residence, smoking, eating habits, taking activities, antidiabetic medication, antihypertensive therapy, fasting plasma glucose [FPG], low-density lipoprotein cholesterol [LDL-C], estimated glomerular filtration rate [eGFR], and serum uric acid [SUA]) showed no interaction between BMI and TG level and SBP (men, *β* = 0.572,* P* = 0.845; women, *β* = 0.122,* P *= 0.923) and DBP (men, *β* = -0.373,* P *= 0.810; women, *β* = 0.272,* P *= 0.828). A binary logistic regression model analysis with adjustment for confounding factors (age, educational level, marital status, current residence, smoking, drinking, eating habits, taking activities, major accidental injury, physical activity, history of cardiovascular disease, history of liver disease, antilipidemic medication, antidiabetic medication, antihypertensive therapy, FPG, LDL-C, high-density lipoprotein cholesterol [HDL-C], eGFR, and SUA) showed that overweight and obese men and women were more likely to have hypertension (men: odds ratio [OR] = 1.781, 95% confidence interval [CI] = 1.393–2.277; women: OR = 1.653, 95% CI = 1.330–2.055) and women with high TG were more likely to have hypertension (OR = 1.558, 95% CI = 1.219–1.992).

**Conclusion:**

An interactive effect of BMI and TG level on BP was not observed in either men or women; however, independent effects of BMI on BP were observed in both men and women, and an association between TG level and hypertension was observed in women.

## 1. Introduction

The prevalence of hypertension has dramatically increased in the past few years in China[1, 2]; furthermore, the rate of hypertension is disproportionately high among elderly individuals in this country[3–5]. Hypertension is defined as a diastolic blood pressure (DBP) of ≥ 90 mmHg and/or systolic blood pressure (SBP) of ≥ 140 mmHg based on the evidence of modestly increasing risk[6–8] and incidence of cardiovascular disease (CVD)[9–11].

Hypertension is a complex disease, and patients with the condition suffer from an economic, psychosocial, and physical burden. Recently, hypertension has become an important global public health challenge [12]. Therefore, an effective strategy to prevent hypertension and determine its associated risks should be carefully implemented. Numerous studies[13–20] have been conducted to determine the risk factors for hypertension, such as aging, overweight, central obesity, lifestyle, family history of hypertension, history of diabetes and dyslipidemia, lack of physical activity, smoking, alcohol consumption, low-density lipoprotein cholesterol (LDL-C) level, triglycerides (TG) level, and high-density lipoprotein cholesterol (HDL-C) level. Lipid abnormalities have been shown to increase the risk factors for hypertension [21]. For example, Teng [22] found an additive effect of TG on DBP. Brennan [23] conducted a study to investigate the effects of body weight on TG and SBP in individuals aged 20 to 49 years and found that, in men, significant correlations with BMI were found for all variables, while in women younger than 40, only the correlation with BP was significant. TG level has been associated with BP since TG level itself can lead to endothelial dysfunction [24, 25], arterial stiffness [26], and loss of vasomotor reactivity [27]. However, it is also important to understand the interrelationships among various risk factors for hypertension. In particular, the association between TG level and risk of hypertension and the effects of obesity on this association are of considerable interest, and an interactive effect between body mass index (BMI) and TG level on BP may also be considered.

No consistent recognition of the association and interaction analysis between BMI and TG level and BP in elderly individuals exists. Thus, the present study particularly aimed to determine the prevalence of normotension and hypertension and their association with BMI, TG level, and other confounding factors based on sex, using cross-sectional data from community-dwelling individuals aged ≥ 60 years in China.

## 2. Methods

### 2.1. Study Design and Setting

Data for this cross-sectional study were taken from the China Health and Retirement Longitudinal Study (CHARLS), a biennial and nationally representative longitudinal survey conducted by the China Centre for Economic Research at Peking University [28]. The baseline survey had a four-stage, stratified, cluster probability sampling design.

In the first stage, all counties in China were stratified by region, rural/urban status, and gross domestic product per capita. A random sample of 150 counties was selected to represent the socioeconomic and geographic pattern of all the counties. In the second stage, three primary sampling units (PSUs) were selected in each county with the probability of inclusion of each county in the sample proportional to their population size. In the third stage, all households in each selected PSU were mapped, and a random sample of 24 households was selected among all the households with residents aged ≥ 45 years within each PSU. Finally, for each selected household, one resident aged ≥ 45 years was randomly selected as a participant in the survey. From the 2011 CHARLS Wave1, we included a total of 3629 individuals in our study.

### 2.2. Information on Demographic Characteristics and Self-Reported Risk Factors

Data including age, education, marital status, current residence, smoking, drinking, eating habits, taking activities, accidental injury, physical exercise, history of cardiovascular disease, history of liver disease, antilipidemic medication, antidiabetic medication, and antihypertensive therapy were obtained using a self-reported questionnaire. (1) Median age was 68 years, and age was categorized as < 68 years and ≥ 68 years. (2) Educational levels were classified into illiterate, less than elementary school, high school, and above vocational school. (3) Marital status was classified into married and single. (4) Current residence was classified into rural and urban. (5) Smoking status was never smoked, ex-smoker, and current smoker. (6) Alcohol consumption was classified into more than non-drinker, less than once a month, and once a month. (7) Eating habits were categorized into 2 meals per day or fewer, 3 meals per day, and 4 meals per day or more. (8) Activity status was dichotomized into at least once a month versus never. (9) Major accidental injury information was obtained by asking the participant whether he/she suffered from any type of major accidental injury and received medical treatment; the answer was “yes” or “no.” (10) Regular physical exercise was defined as exercising at least 3 days per week and more than 30 minutes per day, including moderate to vigorous physical activity and walking. (11) History of CVD, history of liver disease, antilipidemic medication, antidiabetic medication, and antihypertensive therapy were defined as a history of receiving treatment for the respective diseases; the answer was “yes” or “no.”

### 2.3. Glucose, LDL, HDL, Triglycerides, eGFR, BP, and Uric Acid Measurement

Venous blood samples were obtained at the Centers for Disease Control and Prevention (CDC) station, then immediately stored and frozen at −20°C, and transported within 2 weeks to the Chinese CDC in Beijing, where they were placed in a deep freezer and stored at −80°C until the relevant assay was performed at the China Medical University laboratory. (1) Fasting plasma glucose (FPG), LDL, HDL, and TG levels were analyzed at the Youanmen Center for Clinical Laboratory at Capital Medical University using the enzymatic colorimetric tests, and serum uric acid (SUA) levels were analyzed using the urinalysis (UA) plus method. We classified TG levels into 2 categories: ≥ 150 mg/dL and < 150 mg/dL, a categorization widely used in previous studies [29, 30]. (2) BP was measured 3 times at intervals of 45 seconds with a sphygmomanometer. The value of BP was determined based on the mean of the 3 measurements. Normotension was defined as absence of antihypertensive therapy with an SBP of < 140 mmHg and DBP of < 90 mmHg, while hypertension was defined as an SBP of ≥140 mmHg and/or DBP of ≥ 90 mmHg; this categorization has been widely used in previous studies. (3) Estimated glomerular filtration rate (eGFR) was calculated using the CKD-EPI creatinine-cystatin equations [31]: (1) in men, serum creatinine (Scr) ≤ 0.9, serum cystatin C(Scys) ≤ 0.8, eGFR = 135*∗* (Scr/0.9)^−0.207^*∗* (Scys/0.8)^−0.375^*∗*0.995^age^; Scr≤ 0.9, Scys> 0.8, eGFR = 135*∗* (Scr/0.9)^−0.207^*∗* (Scys/0.8)^−0.711^*∗*0.995^age^; Scr> 0.9, Scys≤ 0.8, eGFR=135*∗* (Scr/0.9)^−0.601^*∗* (Scys/0.8)^−0.375^*∗*0.995^age^; Scr> 0.9, Scys> 0.8, eGFR = 135*∗* (Scr/0.9)^−0.601^*∗* (Scys/0.8)^−0.711^*∗*0.995^age^; (2) in women, Scr ≤ 0.7, Scys ≤ 0.8, eGFR = 130*∗*(Scr/0.7)^−0.248^*∗*(Scys/0.8)^−0.375^*∗*0.995^age^; Scr≤ 0.7, Scys> 0.8, and eGFR = 130*∗*(Scr/0.7)^−0.248^*∗*(Scys/0.8)^−0.711^*∗*0.995^age^; Scr> 0.7, Scys≤ 0.8, eGFR = 130*∗* (Scr/0.7)^−0.601^*∗*(Scys/0.8)^−0.375^*∗*0.995^age^; Scr> 0.7, Scys> 0.8, and eGFR = 130*∗* (Scr/0.7)^−0.601^*∗* (Scys/0.8)^−0.711^*∗*0.995^age^.

### 2.4. Measurement of Body Mass Index

Weight and height were measured using a weight and height measurement instrument. BMI was calculated based on the measured weight and height of the participants, who were classified into four categories: underweight (BMI, ≤ 18.5 kg/m^2^), normal weight (18.5–24 kg/m^2^), overweight (24–28 kg/m^2^), and obese (> 28 kg/m^2^)[32].

### 2.5. Statistical Analysis

Our data are represented as mean ± standard deviation (SD; continuous data) and number and percentage (categorical data). Differences between normotension and hypertension, or between normal weight individuals and those with underweight or adiposity, were evaluated using the t-test or chi-square test, followed by Bonferroni adjustment. Correlations between various characteristics and BP were compared using the age-adjusted partial Pearson's correlation test. The adjusted associations between various characteristics and BP were first compared using linear regression models, as appropriate. Then, general linear models adjusted for related potential confounders (age, educational level, marital status, current residence, smoking, drinking, eating habits, taking activities, major accidental injury, physical activity, history of CVD, history of liver disease, antilipidemic medication, antidiabetic medication, antihypertensive therapy, FPG, LDL-C, HDL-C, eGFR, and SUA) [33] were constructed to examine the synergistic effect of BMI and TG level on BP. A binary logistic regression model adjusted for related potential confounders was used to examine the association between BMI or TG and hypertension. A* P*-value of 0.05 was considered significant. All data were analyzed using the SPSS version 17.0 (IBM Corp., Armonk, NY, USA).

### 2.6. Patient and Public Involvement Statement

Consent from the respondents was obtained by the CHARLS. The data information obtained from the study was public, and patients were not involved.

## 3. Results

The 2011 CHARLS Wave1 sample (N=3629) consists of 49.88% men (Age: Mean = 68.86 years; SD = 6.30; range, 60–93 years) and 50.12% women (Age: Mean = 68.55 years; SD = 6.82; range, 60–96 years). Among men, the mean SBP and DBP were 128.93 mmHg and 72.28 mmHg, respectively, and among women, the mean SBP and DBP were 133.66 mmHg and 73.00 mmHg, respectively. In men, 10.94%, 60.66%, 21.60%, and 6.80% were underweight, normal weight, overweight, and obese, respectively, whereas in women 10.34%, 49.04%, 29.08%, and 11.54% were underweight, normal weight, overweight, and obese, respectively. The mean and SD of TG level were 112.79 ± 83.55 mg/dL in men and 140.14 ± 94.12 mg/dL in women. Tables 1 and 2 present the baseline characteristics of the sample for all variables, and most variables were based on our previous research [32].

Tables 1 and 2 show the various characteristics of participants categorized on basis of BMI. The participants comprised 1,810 men aged 68.86 ± 6.30 (range: 60–93) years and 1,819 women aged 68.55 ± 6.82 (range, 60–96) years. According to the modified Chinese criteria for BMI [34], the mean BMI in men was 22.41 kg/m^2^ (SD, 3.71), with 10.94% underweight (BMI, < 18.5 kg/m^2^), 60.66% normal weight (BMI, 18.5–24 kg/m^2^), 21.60% overweight, and 6.80% obese (BMI, ≥ 28 kg/m^2^), whereas the mean BMI in women was 23.33 kg/m^2^ (SD, 4.23), with 10.34% underweight (BMI, < 18.5 kg/m^2^), 49.04% normal weight (BMI, 18.5–24 kg/m^2^), 29.08% overweight, and 11.54% obese (BMI, ≥ 28 kg/m^2^).  Table 1 shows the background characteristics of male participants categorized based on BMI. Levels of FPG, LDL-C, TG, eGFR, SUA, SBP, and DBP were significantly higher in the high BMI group than in the low BMI group, whereas HDL-C level was higher in the low BMI group than in the high BMI group. However, between-group differences in the prevalence of major accidental injury, regular physical exercise, history of liver disease, and antihypertensive therapy were not observed. In women, levels of FBG, LDL-C, TG, eGFR, SUA, SBP, and DBP were significantly higher in the high BMI group than those in the low BMI group, but HDL-C level was higher in the low BMI group than in the high BMI group. However, between-group differences in categories of alcohol drinking, major accidental injury, physical activity, and history of liver disease were not observed (Table 2).

Tables 3 and 4 show the characteristics of participants categorized by BMI and BP status. First, in the hypertensive group with a BMI < 24.0 kg/m^2^ in men, levels of glucose, LDL-C, BMI, SUA, SBP, and DBP were significantly higher than those in the hypertensive group, but eGFR level was significantly higher than that in the normotensive group. Second, in the hypertensive group with a BMI ≥ 24.0 kg/m^2^ in men, levels of SUA, SBP, and DBP were also significantly higher, but eGFR level was significantly lower than that in the normotensive group (shown in Table 3). Third, in the hypertensive group with a BMI < 24.0 kg/m^2^ in women, levels of TG, SUA, SBP, DBP, and the prevalence of antilipidemic medication were significantly higher than those in the normotensive group, but eGFR level was significantly lower. Lastly, in the hypertensive group with a BMI ≥ 24.0 kg/m^2^ in women, the prevalence of CVD, antilipidemic medication, and antidiabetic medication were significantly higher, as were levels of glucose, TG, SBP, and DBP (Table 4).

Table 5 shows the various characteristics of participants categorized by age. SUA and SBP levels were significantly higher in the older age group than in the younger age group in men, whereas levels of HDL-C, eGFR, DBP, BMI, and TG were lower in the older age group than in the younger age group. However, between-group differences in categories of current residence, eating habits, taking activities, major accidental injury, regular physical exercise, history of liver disease, antidiabetic medication, antihypertensive therapy, FPG, and LDL-C were not observed. In women, SUA and SBP levels were significantly higher in the older age group than in the younger age group, but eGFR and BMI levels were lower in the older age group than in the younger age group. However, between-group differences in categories of current residence, drinking, eating habits, activity, major accidental injury, history of CVD, history of liver disease, antidiabetic medication, and antihypertensive therapy, FPG, LDL-C, HDL-C, DBP, and TG were not observed.

**Figure 1:**
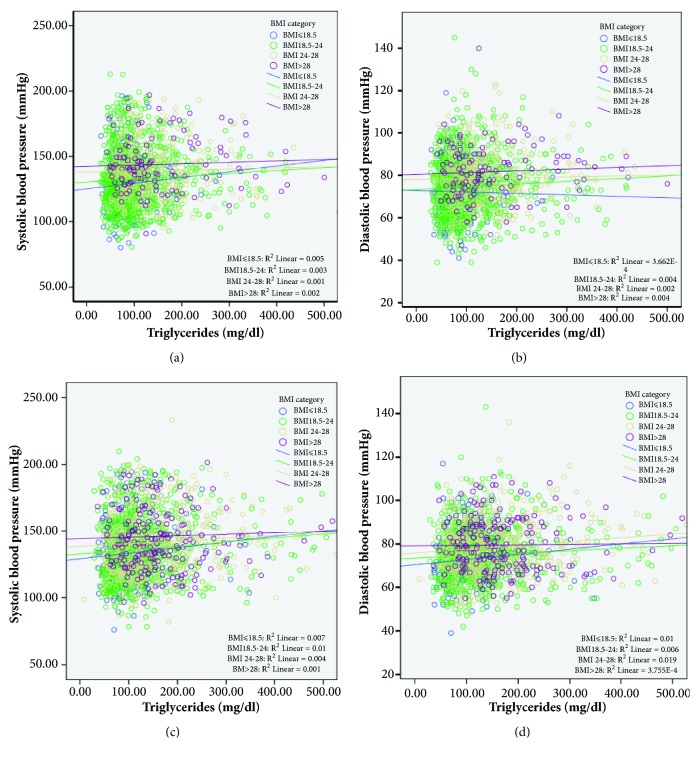
(a, b) Correlation between serum uric acid and blood pressure status of participants categorized by body mass index in male. (c, d) Correlation between triglycerides and blood pressure status of participants categorized by body mass index in female.

In addition to their direct associations, we observed the effect between BMI category and TG levels on BP in Figure 1. TG correlated positively with both SBP and DBP. Analysis of covariance showed that three regression lines in each graph were not different from those in the other groups (male: SBP, F=0.028,* P*=0.867 and DBP, F= 0.194, and* P* =0.660; female: SBP, F=0.783,* P*=0.376 and DBP, F=0.005, and* P* =0.941; respectively).

Tables 6 and 7 show the relationship between various characteristics and BP status of participants categorized by BMI (< 24.0 kg/m^2^ and ≥ 24.0 kg/m^2^). Age-adjusted partial Pearson's correlation coefficient showed that TG level was positively correlated with both SBP and DBP in men with a BMI < 21.0 kg/m^2^ (Table 6). In women, the TG level was significantly correlated with SBP and DBP in subjects with a BMI < 21.0 kg/m^2^ and positively correlated with DBP in those with a BMI ≥ 24.0 kg/m^2^, but there was no correlation with SBP in women with a BMI ≥ 24.0 kg/m^2^ (Table 7).

Tables 8 and 9 show the relationship between various characteristics and BP status of participants categorized by age (< 68 years and ≥ 68 years). TG level was positively correlated with both SBP and DBP in both men and women with age < 68 years and ≥ 68 years.

Tables 10 and 11 show the multivariate-adjusted relationship between various characteristics and BP status in participants categorized by BMI (< 24.0 kg/m^2^ and ≥ 24.0 kg/m^2^). Multiple linear regression analysis showed that TG level was significantly and positively associated with SBP in women with a BMI < 24.0 kg/m^2^, independent of other confounding factors; however, TG level was not significantly associated with BP in men.

Tables 12 and 13 show the multivariate-adjusted relationship between various characteristics and BP status in participants categorized by age (< 68 years and ≥ 68 years). Multiple linear regression analysis showed that TG level was significantly and positively associated with SBP in women with age of < 68 years, independent of other confounding factors. In contrast, TG level was significantly associated with SBP in men with age of ≥ 68 years.

Table 14 shows the interaction between BMI and TG level and BP status in men and women. A general linear model with the following confounding factors (age, educational level, marital status, current residence, smoking, eating habits, taking activities, antidiabetic medication, antihypertensive therapy, FPG, LDL-C, eGFR, and SUA) was used to assess the statistical significance of the synergistic relationship between BMI and SUA level. Evidence of interaction between BMI and SUA level on SBP (men, *β* = 0.572,* P* = 0.845; women, *β* = 0.122, and* P *= 0.923) and DBP (men, *β* = -0.373,* P *= 0.810; women, *β* = 0.272, and* P *= 0.828) levels was not observed.

Table 15 shows the interaction between BMI and TG on BP status of participants categorized by age in men and women. A general linear model with the following confounding factors (age, educational level, marital status, current residence, smoking, eating habits, activity, physical activity, antilipidemic medication, antidiabetic medication, antihypertensive therapy, FPG, eGFR, and SUA) was used to assess the statistical significance of the synergistic relationship between BMI and SUA level. Evidence of interaction between BMI and SUA level on SBP (men with age of < 68 years, *β* = -1.075, and* P* = 0.760; men with age of ≥ 68 years, *β* = 2.138, and* P* = 0.654; women with age of < 68 years, *β* = -1.345, and* P *= 0.718; women with age of ≥ 68 years, *β* = -4.192, and* P* = 0.334) and DBP (men with age of < 68 years, *β* = -0.095, and* P* = 0.964; men with age of ≥ 68 years, *β* = -0.931, and* P* = 0.691; women with age of < 68 years, *β* = 2.410,* P *= 0.170; women with age of ≥ 68 years *β* = -3.386, and* P* = 0.439) was not observed.

Table 16 shows relationships between BMI or TG level and hypertension in men and women. After adjusting for age, educational level, marital status, current residence, smoking, drinking, eating habits, activity, major accidental injury, physical activity, history of CVD, history of liver disease, antilipidemic medication, antidiabetic medication, antihypertensive therapy, FPG, LDL-C, HDL-C, eGFR, and SUA, compared with their counterparts with BMI < 24.0 kg/m^2^, both elderly men and women with a BMI ≥ 24.0 kg/m^2^ were more likely to have high BP (men: odds ratio [OR] = 1.781, 95% confidence interval [CI] = 1.393–2.277; women: OR = 1.653, 95% CI = 1.330–2.055). Among women, compared to those with TG < 150 mg/dL, individuals with a high TG level were more likely to have high BP (OR = 1.558, 95% CI = 1.219–1.992).

Tables 17 and 18 show relationships between BMI or TG and hypertension categorized by age in men and women. After adjusting for age, educational level, marital status, current residence, smoking, drinking, eating habits, taking activities, major accidental injury, physical activity, history of CVD, history of liver disease, antilipidemic medication, antidiabetic medication, antihypertensive therapy, FPG, LDL-C, HDL-C, eGFR, and SUA, compared to individuals with a BMI < 24.0 kg/m^2^, both elderly men and women with a BMI ≥ 24.0 kg/m^2^ were more likely to have high BP (men with age of < 68 years, OR=1.805, and 95% CI=1.249-2.610; men with age of ≥ 68 years, OR = 1.796, and 95% CI = 1.275–2.529; women with age of < 68 years, OR = 1.936, and 95% CI = 1.404–2.668; women with age of ≥ 68 years, OR = 1.506, and 95% CI = 1.108–2.047). Among women, compared to those with TG < 150 mg/dL, individuals with a high TG level were more likely to have high BP (women with age of < 68 years, OR = 1.629, and 95% CI = 1.149–2.309; women with age of ≥ 68 years, OR = 1.596, and 95% CI = 1.113–2.288).

## 4. Discussion

The effects of BMI and TG level on BP varied in elderly individuals. In the present study, we determined the prevalence of hypertension and its association with BMI and TG level. Our findings show that the prevalence of hypertension was 36.91% (668/1810) in men, 43.82% (797/1819) in women, and 40.37% (1465/3629) overall. After stratification by age, the prevalence of hypertension was 39.20% (481/1227) in men (age ≥ 65 years), 49.91% (576/1154) in women (age ≥ 65 years), and 44.39% (1057/2381) overall. In a sample collected from the Korea National Health and Nutrition Examination Survey (2007) in the elderly Korean population (age ≥ 65 years)[35], the prevalence of hypertension was 62.0%; the prevalence of hypertension in the Japan Gerontological Evaluation Survey[36], conducted in a population aged 65 or older, was 59.54%. The prevalence of hypertension in our study was lower than that observed in the Korean and Japanese studies. Moreover, men with hypertension had a higher prevalence of risk factors, such as age, marital status, eating habits, FPG, LDL-C, TG, eGFR, BMI, and SUA level, than those with normotension. Age, marital status, antilipidemic medication, FPG, TG, eGFR, and SUA level were significantly associated with BP among women. Furthermore, TG levels were positively associated with SBP and DBP in men with BMI < 24 kg/m^2^; however, TG levels were positively associated with both SBP and DBP in women with a BMI < 24 kg/m^2^ and also positively associated with DBP in women with a BMI ≥ 24 kg/m^2^.

Studies [37, 38] have explored the association and/or interaction analysis between BMI and SUA level and BP. Lyngdoh et al. [37] reported that adiposity substantially decreased the association between SUA level and BP in young adults, and BP was independently associated with SUA level in women. Kawamoto et al. [38] concluded that BMI changes the association between SUA level and BP status among community-dwelling men. In other studies [39, 40], the association of TG and SUA levels has been persistent after full adjustment in a multiple logistic model, suggesting that TG levels correlate independently with SUA level, with TG levels having the most influence on SUA. However, there were no previous studies on the interactive effect between BMI and TG level on BP.

As our general linear models adjusting for 13 related potential confounders showed, interaction between BMI and TG on BP was not observed. In our investigation of the relationships between BMI or TG and hypertension, we made several observations. First, we found that overweight and obese men and women were more likely to have hypertension. We also observed that women with high TG were more likely to experience hypertension. Thirdly, no association between TG and hypertension was found in men. Lastly, the relative results in relations between BMI or TG and hypertension categorized by age in both men and women are the same as those above. Furthermore, age differences in relations between BMI or TG and hypertension were found. Specifically, individuals with age ≥ 68 years experienced lower effects of BMI or TG level on BP. Similar evidence has been found in Brazil, in a study based on 287 men and women aged between 18 and 88 years, in which Pimenta [41] found that central obesity and TG level were independent risk factors for hypertension according to multivariate analysis. However, several studies have reported divergent findings regarding the association between obesity and hypertension. Some studies have suggested an increased risk of hypertension with higher BMI, waist circumference (WC), and waist-to-hip ratio (WHR) [42, 43], whereas other studies found that the association was not significant [44–46]. Zhou [47] conducted the first meta-analysis of cohort studies to quantify the relationship between obesity and the incidence of hypertension and found a positive association between the risk of hypertension and BMI, WC, and WHR. Arabshahi [48] conducted the first meta-analysis of cross-sectional studies to investigate the relationship between BMI or WC and hypertension, and concluded that the risk of hypertension was associated with adiposity. Jayedi [49] conducted a meta-analysis of prospective cohort studies to report the risk estimates of hypertension for abdominal adiposity (BMI, WC, and WHR) and found that the risk of hypertension increased with a somewhat steeper trend with increasing BMI, in comparison with WC and WHR. Such discrepancies between our findings and the null studies may be a result of methodological differences in design, measurement of obesity, and populations. Moreover, TG level has been associated with BP since TG level itself can cause endothelial dysfunction [24, 25], arterial stiffness [26], and the loss of vasomotor reactivity [27]. Such pathophysiology induced by increased TG and FPG levels and low HDL-C level may be greater than that of SUA. To explore the extent of the effects of TG level on blood pressure, we controlled for SUA and the related confounders, such as LDL-C and HDL-C [38]. The findings showed that TG level and hypertension were observed in women, but no independent effect was observed in men. Sanchez-Inigo [50] conducted a cohort study to identify the association of TG with the incidence of hypertension in Spain and found that the incidence of hypertension was associated with TG level in both men and women independent of adiposity. Tohidi [51] found that high TG independently predicted incidence of hypertension in Middle Eastern women.

The mechanisms that lead to hypertension in participants with high BMI or TG levels have not been completely understood. Current studies may provide insight into the pathogenic mechanisms of BMI/TG that induce hypertension. The present study suggests that TG level may play an important role in hypertension in women. We speculate that sex-specific factors may also play an important role. TG levels are higher in women than in men, which partially explains the underlying mechanism that accounts for sex difference based on hormone levels. Additionally, body fat, sex steroids, and their interaction in elderly participants may also be associated with hypertension. As an important sex hormone, estrogen may also affect BP. Elderly women have lower concentrations of estrogen, which may affect the level of TG and result in a smaller protective effect.

## 5. Strengths and Limitations of the Study

There are several limitations of our study. First, the association and interaction of BMI and serum TG on blood pressure become seriously more complex; we only consider the confounders as possible as we can, but there are some unknown factors. Secondly, the relationship should be studied prospectively. Our study investigated BP in the elderly participant through a cross-sectional study. Follow-up study was relatively short to comprehensively observe changes in the next step. Last, more research is needed to observe the result. Several strengths could be found in our study. Firstly, the study was based on a nationwide survey. Secondly, we conducted the analyses according to gender.

## 6. Conclusions

An interactive effect of BMI and TG level on BP was not observed in either men or women; however, independent effects of BMI on BP were observed in both men and women, and an association between TG level and hypertension was observed in women.

## Figures and Tables

**Table 1:** Various characteristics of participants categorized by BMI in male (N=1810).

**Variables**	BMI≤18.5 (n=198)	BMI18.5-24 (n=1098)	BMI 24-28 (n=391)	BMI>28 (n=123)	t/*χ*^2^	***P***
Age(years)	71.49±6.31	69.17±6.4	67.35±5.71	66.7±5.22	25.817	<0.001
Education						
Illiterate	52(26.26)	256(23.32)	44(11.25)	20(16.26)	42.976	<0.001
Less than elementary school	130(65.66)	773(70.40)	306(78.26)	90(73.17)		
High school	6(3.03)	17(1.55)	5(1.28)	4(3.25)		
Above vocational school	10(5.05)	52(4.74)	36(9.21)	9(7.32)		
Marital status						
Single	29(14.65)	179(16.30)	30(7.67)	13(10.57)	19.2333	<0.001
Married	169(85.35)	919(83.70)	361(92.33)	110(89.43)		
Current residence						
Rural	152(76.77)	791(72.04)	213(54.48)	65(52.85)	60.303	<0.001
Urban	46(23.23)	307(27.96)	178(45.52)	58(47.15)		
Smoke						
NO	118(59.60)	632(57.56)	163(41.69)	48(39.02)	43.161	<0.001
Former smoke	36(18.18)	199(18.12)	105(26.85)	33(26.83)		
Current smoke	44(22.22)	267(24.32)	123(31.46)	42(34.15)		
Drinking						
NO	115(58.08)	543(49.45)	202(51.66)	65(52.85)	197.72	<0.001
Less than once a month	13(6.57)	94(8.56)	33(8.44)	14(11.38)		
More than once a month	70(35.35)	461(41.99)	156(39.9)	44(35.77)		
Eating meals						
≤2 meals per day	31(15.66)	174(15.85)	43(11.00)	10(8.13)	15.580	0.016
3 meals per day	162(81.82)	905(82.42)	345(88.24)	113(91.87)		
≥4 meals per day	5(2.53)	19(1.73)	3(0.77)	0(0.00)		
Taking activities						
No	119(60.10)	593(54.01)	175(44.76)	62(50.41)	15.198	0.002
Yes	79(39.90)	505(45.99)	216(55.24)	61(49.59)		
Ever been in major accidental injury						
No	173(87.37)	976(88.89)	349(89.26)	111(90.24)	0.743	0.863
Yes	25(12.63)	122(11.11)	42(10.74)	12(9.76)		
Having regular physical exercises						
No physical exercise	122(61.62)	701(63.84)	247(63.17)	69(56.1)	5.204	0.518
Less than regular physical exercises	39(19.70)	183(16.67)	60(15.35)	23(18.70)		
Regular physical exercises	37(18.69)	214(19.49)	84(21.48)	31(25.20)		
History of CVD						
No	178(89.90)	965(87.89)	323(82.61)	87(70.73)	33.007	<0.001
Yes	20(10.10)	133(12.11)	68(17.39)	36(29.27)		
History of liver diseases						
No	194(97.98)	1055(96.08)	375(95.91)	114(92.68)	5.576	0.134
Yes	4(2.02)	43(3.92)	16(4.09)	9(7.32)		
Antilipidemic medication						
No	194(97.98)	1063(96.81)	366(93.61)	104(84.55)	43.969	<0.001
Yes	4(2.02)	35(3.19)	25(6.39)	19(15.45)		
Anti-diabetic medication						
No	195(98.48)	1069(97.36)	365(93.35)	104(84.55)	54.001	<0.001
Yes	3(1.52)	29(2.64)	26(6.65)	19(15.45)		
Anti-hypertensive therapy						
No	186(93.94)	1036(94.35)	359(91.82)	114(92.68)	3.345	0.341
Yes	12(6.06)	62(5.65)	32(8.18)	9(7.32)		
Fasting plasma glucose(mg/dl)	105.00±28.94	109.16±36.00	118.37±41.50	119.64±41.26	9.994	<0.001
LDL Cholesterol (mg/dl)	105.37±32.12	110.54±32.35	118.02±35.30	119.42±34.05	9.701	<0.001
HDL Cholesterol (mg/dl)	60.04±16.14	53.83±15.90	44.00±13.320	42.9±12.28	74.457	<0.001
eGFR(ml/min/1.73m^2^)	71.56±16.22	74.92±16.53	75.57±16.32	75.13±16.23	2.868	0.035
Serum uric acid(mg/dl)	4.79±1.29	4.98±1.31	5.37±1.34	5.27±1.29	12.990	<0.001
Systolic blood pressure(mmHg)	128.93±21.56	132.59±24.29	139.25±25.58	143.94±19.85	23.845	<0.001
Diastolic blood pressure(mmHg)	72.28±12.34	74.59±12.78	78.65±12.37	81.69±12.29	17.079	<0.001
Body mass index(kg/m^2^)	17.24±0.90	21.25±1.53	25.65±1.12	30.71±4.3.00	2126.849	<0.001
Triglycerides (mg/dl)	84.34±34.71	101.52±59.33	146.98±129.67	150.5±92.37	47.962	<0.001

**Table 2:** Various characteristics of participants categorized by BMI in female (N=1819).

**Variables**	BMI≤18.5 (n=188)	BMI18.5-24 (n=892)	BMI 24-28 (n=529)	BMI>28 (n=210)	t/*χ*^2^	***P***
Age (years)	71.41±7.07	68.77±6.99	67.64±6.32	67.34±6.25	17.169	<0.001
Education						
Illiterate	133(70.74)	530(59.42)	269(50.85)	111(52.86)	35.807	<0.001
Less than elementary school	55(29.26)	342(38.34)	236(44.61)	97(46.19)		
High school	0(0.00)	6(0.67)	9(1.70)	0(0.00)		
Above vocational school	0(0.00)	14(1.57)	15(2.84)	2(0.95)		
Marital status						
Single	72(38.30)	291(32.62)	131(24.76)	49(23.33)	20.478	<0.001
Married	116(61.70)	601(67.38)	398(75.24)	161(76.67)		
Current residence						
Rural	150(79.79)	588(65.92)	309(58.41)	107(50.95)	43.911	<0.001
Urban	38(20.21)	304(34.08)	220(41.59)	103(49.05)		
Smoke						
NO	151(80.32)	783(87.78)	479(90.55)	190(90.48)	20.377	<0.001
Former smoke	6(3.19)	30(3.36)	15(2.84)	8(3.81)		
Current smoke	31(16.49)	79(8.86)	35(6.62)	12(5.71)		
Drinking						
NO	164(87.23)	781(87.56)	462(87.33)	194(92.38)	7.632	0.266
Less than once a month	10(5.32)	32(3.59)	27(5.10)	6(2.86)		
More than once a month	14(7.45)	79(8.86)	40(7.56)	10(4.76)		
Eating meals						
≤2 meals per day	48(25.53)	143(16.03)	58(10.96)	18(8.57)	32.785	<0.001
3 meals per day	139(73.94)	732(82.06)	463(87.52)	189(90.00)		
≥4 meals per day	1(0.53)	17(1.91)	8(1.51)	3(1.43)		
Taking activities						
No	113(60.11)	490(54.93)	241(45.56)	100(47.62)	19.191	<0.001
Yes	75(39.89)	402(45.07)	288(54.44)	110(52.38)		
Ever been in major accidental injury						
No	173(92.02)	830(93.05)	490(92.63)	197(93.81)	0.573	0.903
Yes	15(7.98)	62(6.95)	39(7.37)	13(6.19)		
Having regular physical exercises						
No physical exercise	134(71.28)	569(63.79)	322(60.87)	132(62.86)	9.032	0.172
Less than regular physical exercises	25(13.30)	172(19.28)	99(18.71)	36(17.14)		
Regular physical exercises	29(15.43)	151(16.93)	108(20.42)	42(20.00)		
History of CVD						
No	160(85.11)	757(84.87)	428(80.91)	158(75.24)	13.931	0.003
Yes	28(14.89)	135(15.13)	101(19.09)	52(24.76)		
History of liver diseases						
No	183(97.34)	864(96.86)	504(95.27)	203(96.67)	3.289	0.349
Yes	5(2.66)	28(3.14)	25(4.73)	7(3.33)		
Antilipidemic medication						
No	185(98.40)	854(95.74)	474(89.60)	178(84.76)	49.744	<0.001
Yes	3(1.60)	38(4.26)	55(10.40)	32(15.24)		
Anti-diabetic medication						
No	187(99.47)	863(96.75)	490(92.63)	188(89.52)	33.154	<0.001
Yes	1(0.53)	29(3.25)	39(7.37)	22(10.48)		
Anti-hypertensive therapy						
No	171(90.96)	838(93.95)	495(93.57)	191(90.95)	33.154	<0.001
Yes	17(9.04)	54(6.05)	34(6.43)	19(9.05)		
Fasting plasma glucose (mg/dl)	105.18±22.29	110.63±40.95	118.34±48.98	116.55±35.01	6.659	<0.001
LDL Cholesterol (mg/dl)	115.41±33.67	122.84±34.65	127.66±38.90	128.45±41.61	6.511	<0.001
HDL Cholesterol (mg/dl)	60.31±16.04	54.55±15.13	47.67±13.02	45.26±11.70	62.326	<0.001
eGFR (ml/min/1.73m^2^)	72.49±16.54	75.97±16.10	77.40±15.8	75.16±18.06	4.396	0.004
Serum uric acid (mg/dl)	3.83±1.14	4.06±1.12	4.30±1.14	4.62±1.22	21.304	<0.001
Systolic blood pressure (mmHg)	133.66±24.74	136.74±23.69	141.83±30.53	146.33±38.15	19.704	<0.001
Diastolic blood pressure (mmHg)	73.00±12.26	75.01±11.77	78.61±12.06	79.54±12.23	10.742	<0.001
Body mass index (kg/m^2^)	17.13±1.42	21.43±1.50	25.73±1.15	30.90±4.33	2202.200	<0.001
Triglycerides (mg/dl)	103.17±50.81	128.46±82.33	158.53±100.30	176.54±128.24	33.113	<0.001

**Table 3:** Characteristics of participants categorized by BMI and blood pressure status in male (N=1810).

Variables	BMI24(n=1296)		t/*χ*^2^	*P*	BMI≥24 (n=514)		t/*χ*^2^	***P***
	Normotension (n=866)	Hypertension (n=430)			Normotension (n=276)	Hypertension (n=238)		
Age(years)	68.99±6.42	70.53±6.33	-4.080	<0.001	66.84±5.47	67.55±5.71	-1.439	0.151
Education								
Illiterate	200(23.09)	108(25.12)	6.278	0.099	37(13.41)	27(11.34)	0.776	0.855
Less than elementary school	600(69.28)	303(70.47)			211(76.45)	185(77.73)		
High school	20(2.31)	3(0.70)			4(1.45)	5(2.10)		
Above vocational school	46(5.31)	16(3.72)			24(8.70)	21(8.82)		
Marital status								
Single	122(14.09)	86(20.00)	7.454	0.006	20(7.25)	23(9.66)	0.974	0.324
Married	744(85.91)	344(80.00)			256(92.75)	215(90.34)		
Current residence								
Rural	638(73.67)	305(70.93)	1.090	0.296	155(56.16)	123(51.68)	1.032	0.310
Urban	228(26.33)	125(29.07)			121(43.84)	115(48.32)		
Smoke								
NO	487(56.24)	263(61.16)	2.980	0.225	103(37.32)	108(45.38)	3.691	0.158
Former smoke	165(19.05)	70(16.28)			81(29.35)	57(23.95)		
Current smoke	214(24.71)	97(22.56)			92(33.33)	73(30.67)		
Drinking								
NO	441(50.92)	217(50.47)	0.051	0.975	139(50.36)	128(53.78)	0.599	0.741
Less than once a month	72(8.31)	35(8.14)			26(9.42)	21(8.82)		
More than once a month	353(40.76)	178(41.4)			111(40.22)	89(37.39)		
Eating meals								
≤2 meals per day	116(13.39)	89(20.70)	11.939	0.003	29(10.51)	24(10.08)	0.233	0.890
3 meals per day	735(84.87)	332(77.21)			245(88.77)	213(89.50)		
≥4 meals per day	15(1.73)	9(2.09)			2(0.72)	1(0.42)		
Taking activities								
No	477(55.08)	235(54.65)	0.021	0.884	120(43.48)	117(49.16)	1.334	0.248
Yes	389(44.92)	195(45.35)			156(56.52)	121(50.84)		
Ever been in major accidental injury								
No	760(87.76)	389(90.47)	2.091	0.148	243(88.04)	217(91.18)	1.741	0.187
Yes	106(12.24)	41(9.53)			33(11.96)	21(8.82)		
Having regular physical exercises								
No physical exercise	563(65.01)	260(60.47)	2.957	0.228	170(61.59)	146(61.34)	1.743	0.418
Less than regular physical exercises	139(16.05)	83(19.30)			49(17.75)	34(14.29)		
Regular physical exercises	164(18.94)	87(20.23)			57(20.65)	58(24.37)		
History of CVD								
No	772(89.15)	376(87.44)	0.947	0.330	222(80.43)	183(76.89)	0.760	0.383
Yes	94(10.85)	54(12.56)			54(19.57)	55(23.11)		
History of liver diseases								
No	832(96.07)	417(96.98)	0.634	0.426	260(94.20)	229(96.22)	1.122	0.290
Yes	34(3.93)	13(3.02)			16(5.80)	9(3.78)		
Antilipidemic medication								
No	839(96.88)	418(97.21)	0.105	0.746	253(91.67)	217(91.18)	0.039	0.843
Yes	27(3.12)	12(2.79)			23(8.33)	21(8.82)		
Anti-diabetic medication								
No	841(97.11)	423(98.37)	1.891	0.169	252(91.30)	217(91.18)	0.003	0.959
Yes	25(2.89)	7(1.63)			24(8.70)	21(8.82)		
Anti-hypertensive therapy								
No	866(100.00)	356(82.79)	154.880	<0.001	276(100.00)	197(82.77)	49.347	<0.001
Yes	0(0.00)	74(17.21)			0(0.00)	41(17.23)		
Fasting plasma glucose(mg/dl)	106.7±33.3	112.26±38.38	-2.678	0.007	116.83±37.59	120.62±45.51	-1.029	0.304
LDL Cholesterol (mg/dl)	108.4±32.03	112.4±33.07	-2.089	0.037	117.46±34.23	119.22±35.95	-0.565	0.572
HDL Cholesterol (mg/dl)	54.94±16.53	54.59±15.34	0.364	0.716	44.02±12.93	43.18±13.00	0.735	0.463
eGFR(ml/min/1.73m^2^)	75.95±15.97	71.69±17.21	4.396	<0.001	77.54±16.00	73.21±16.46	3.011	0.003
Serum uric acid(mg/dl)	4.83±1.25	5.15±1.38	-4.246	<0.001	5.22±1.32	5.49±1.31	-2.278	0.023
Systolic blood pressure(mmHg)	119.36±12.22	157.19±21.46	-25.143	<0.001	124.62±10.54	158.37±23.28	-15.737	<0.001
Diastolic blood pressure(mmHg)	69.02±9.26	84.54±12.44	-40.141	<0.001	72.72±8.41	86.95±11.89	-21.480	<0.001
Body mass index(kg/m^2^)	20.53±2.05	20.84±2.04	-2.604	0.009	26.80±3.76	26.88±2.08	-0.280	0.780
Triglycerides (mg/dl)	95.95±54.77	104.34±59.81	-2.510	0.012	147.99±135.77	147.86±104.59	0.012	0.990

**Table 4:** Characteristics of participants categorized by BMI and blood pressure status in female (N=1819).

Variables	BMI24(n=1080)		t/*χ*^2^	*P*	BMI≥24 (n=739)		t/*χ*^2^	*P*
	Normotension (n=660)	Hypertension (n=420)			Normotension (n=362)	Hypertension (n=377)		
Age(years)	67.87±6.60	71.12±7.30	-7.518	<0.001	66.27±5.54	68.71±6.70	-5.352	<0.001
Education								
Illiterate	385(58.33)	278(66.19)	7.660	0.054	168(46.41)	212(56.23)	11.887	0.008
Less than elementary school	260(39.39)	137(32.62)			178(49.17)	155(41.11)		
High school	5(0.76)	1(0.24)			8(2.21)	1(0.27)		
Above vocational school	10(1.52)	4(0.95)			8(2.21)	9(2.39)		
Marital status								
Single	189(28.64)	174(41.43)	18.823	<0.001	82(22.65)	98(25.99)	1.120	0.290
Married	471(71.36)	246(58.57)			280(77.35)	279(74.01)		
Current residence								
Rural	450(68.18)	288(68.57)	0.018	0.893	207(57.18)	209(55.44)	0.228	0.633
Urban	210(31.82)	132(31.43)			155(42.82)	168(44.56)		
Smoke								
NO	574(86.97)	360(85.71)	0.444	0.801	330(91.16)	339(89.92)	0.925	0.630
Former smoke	22(3.33)	14(3.33)			9(2.49)	14(3.71)		
Current smoke	64(9.70)	46(10.95)			23(6.35)	24(6.37)		
Drinking								
NO	575(87.12)	370(88.10)	1.172	0.556	318(87.85)	338(89.66)	1.871	0.392
Less than once a month	29(4.39)	13(3.10)			20(5.52)	13(3.45)		
More than once a month	56(8.48)	37(8.81)			24(6.63)	26(6.90)		
Eating meals								
≤2 meals per day	112(16.97)	79(18.81)	0.599	0.741	40(11.05)	36(9.55)	0.611	0.737
3 meals per day	537(81.36)	334(79.52)			316(87.29)	336(89.12)		
≥4 meals per day	11(1.67)	7(1.67)			6(1.66)	5(1.33)		
Taking activities								
No	366(55.45)	237(56.43)	0.099	0.753	171(47.24)	170(45.09)	0.342	0.559
Yes	294(44.55)	183(43.57)			191(52.76)	207(54.91)		
Ever been in major accidental injury								
No	610(92.42)	393(93.57)	0.510	0.475	333(91.99)	354(93.90)	1.030	0.310
Yes	50(7.58)	27(6.43)			29(8.01)	23(6.10)		
Having regular physical exercises								
No physical exercise	417(63.18)	286(68.10)	4.349	0.114	223(61.60)	231(61.27)	0.840	0.657
Less than regular physical exercises	133(20.15)	64(15.24)			62(17.13)	73(19.36)		
Regular physical exercises	110(16.67)	70(16.67)			77(21.27)	73(19.36)		
History of CVD								
No	561(85.00)	356(84.76)	<0.001	0.985	293(80.94)	293(77.72)	0.979	0.322
Yes	99(15.00)	64(15.24)			69(19.06)	84(22.28)		
History of liver diseases								
No	640(96.97)	407(96.90)	0.004	0.952	342(94.48)	365(96.82)	1.912	0.167
Yes	20(3.03)	13(3.10)			20(5.52)	12(3.18)		
Antilipidemic medication								
No	641(97.12)	398(94.76)	3.912	0.048	329(90.88)	323(85.68)	4.822	0.028
Yes	19(2.88)	22(5.24)			33(9.12)	54(14.32)		
Anti-diabetic medication								
No	644(97.58)	406(96.67)	0.785	0.375	343(94.75)	335(88.86)	8.466	0.004
Yes	16(2.42)	14(3.33)			19(5.25)	42(11.14)		
Anti-hypertensive therapy								
No	660(100.00)	349(83.10)	116.690	<0.001	362(100.00)	324(85.94)	52.732	<0.001
Yes	0(0.00)	71(16.90)			0(0.00)	53(14.06)		
Fasting plasma glucose(mg/dl)	107.94±38.43	112.33±38.69	-1.816	0.070	111.30±31.47	123.61±54.14	-3.727	<0.001
LDL Cholesterol (mg/dl)	121.38±33.16	121.89±36.31	-0.235	0.814	126.38±37.50	129.47±41.91	-1.046	0.296
HDL Cholesterol (mg/dl)	56.09±15.43	54.71±15.29	1.429	0.153	47.60±12.33	46.48±13.09	1.183	0.237
eGFR(ml/min/1.73m^2^)	77.62±15.26	72.13±17.03	5.486	<0.001	78.07±15.97	75.73±16.73	1.935	0.053
Serum uric acid(mg/dl)	3.92±1.10	4.17±1.16	-3.510	<0.001	4.30±1.12	4.46±1.21	-1.950	0.052
Systolic blood pressure(mmHg)	120.81±12.07	159.88±17.31	-20.347	<0.001	123.15±10.88	161.97±35.55	-17.786	<0.001
Diastolic blood pressure(mmHg)	69.57±8.82	82.44±11.75	-43.348	<0.001	71.98±8.39	85.32±11.53	-19.691	<0.001
Body mass index(kg/m^2^)	20.6±2.20	20.85±2.19	-1.796	0.073	26.97±2.92	27.43±3.84	-1.810	0.071
Triglycerides (mg/dl)	119.69±73.83	130.67±83.99	-2.243	0.025	150.58±87.77	174.89±122.44	-3.067	0.002

**Table 5:** Characteristics of participants categorized by age in male and female (N=3629).

	Male(n=1810)				Female(n=1819)			
Variables	Age <68 years (n=807)	Age≥68 years (n=1003)	t/*χ*^2^	*P*	Age <68 years (n=887)	Age≥68 years (n=932)	t/*χ*^2^	*P*
Education								
Illiterate	94(11.65)	278(27.72)	73.763	<0.001	409(46.11)	634(68.03)	94.734	<0.001
Less than elementary school	652(80.79)	647(64.51)			457(51.52)	273(29.29)		
High school	16(1.98)	16(1.60)			8(0.90)	7(0.75)		
Above vocational school	45(5.58)	62(6.18)			13(1.47)	18(1.93)		
Marital status								
Single	69(8.55)	182(18.15)	34.471	<0.001	138(15.56)	405(43.45)	168.901	<0.001
Married	738(91.45)	821(81.85)			749(84.44)	527(56.55)		
Current residence								
Rural	538(66.67)	683(68.10)	0.416	0.519	580(65.39)	574(61.59)	2.831	0.092
Urban	269(33.33)	320(31.90)			307(34.61)	358(38.41)		
Smoke								
NO	201(24.91)	275(27.42)	10.454	0.005	791(89.18)	812(87.12)	13.584	0.001
Former smoke	145(17.97)	228(22.73)			15(1.69)	44(4.72)		
Current smoke	461(57.13)	500(49.85)			81(9.13)	76(8.15)		
Drinking								
NO	366(45.35)	559(55.73)	19.444	<0.001	777(87.6)	824(88.41)	1.095	0.578
Less than once a month	79(9.79)	75(7.48)			41(4.62)	34(3.65)		
More than once a month	362(44.86)	369(36.79)			69(7.78)	74(7.94)		
Eating meals								
≤2 meals per day	107(13.26)	151(15.05)	1.687	0.430	119(13.42)	148(15.88)	2.586	0.275
3 meals per day	686(85.01)	839(83.65)			752(84.78)	771(82.73)		
≥4 meals per day	14(1.73)	13(1.3)			16(1.8)	13(1.39)		
Taking activities								
No	412(51.05)	537(53.54)	1.108	0.292	448(50.51)	496(53.22)	1.339	0.247
Yes	395(48.95)	466(46.46)			439(49.49)	436(46.78)		
Ever been in major accidental injury								
No	723(89.59)	886(88.33)	0.715	0.398	825(93.01)	865(92.81)	0.027	0.869
Yes	84(10.41)	117(11.67)			62(6.99)	67(7.19)		
Having regular physical exercises								
No physical exercise	509(63.07)	630(62.81)	3.842	0.146	530(59.75)	627(67.27)	11.203	0.004
Less than regular physical exercises	148(18.34)	157(15.65)			181(20.41)	151(16.2)		
Regular physical exercises	150(18.59)	216(21.54)			176(19.84)	154(16.52)		
History of CVD								
No	712(88.23)	841(83.85)	6.902	0.009	741(83.54)	762(81.76)	0.977	0.323
Yes	95(11.77)	162(16.15)			146(16.46)	170(18.24)		
History of liver diseases								
No	769(95.29)	969(96.61)	2.154	0.142	858(96.73)	896(96.14)	0.450	0.503
Yes	38(4.71)	34(3.39)			29(3.27)	36(3.86)		
Antilipidemic medication								
No	759(94.05)	968(96.51)	6.177	0.013	811(91.43)	880(94.42)	6.206	0.013
Yes	48(5.95)	35(3.49)			76(8.57)	52(5.58)		
Anti-diabetic medication								
No	769(95.29)	964(96.11)	0.739	0.390	838(94.48)	890(95.49)	0.911	0.320
Yes	38(4.71)	39(3.89)			49(5.52)	42(4.51)		
Anti-hypertensive therapy								
No	755(93.56)	940(93.72)	0.002	0.888	823(92.78)	872(93.56)	0.433	0.511
Yes	52(6.44)	63(6.28)			64(7.22)	60(6.44)		
Fasting plasma glucose(mg/dl)	113.22±40.77	109.94±34.06	1.864	0.062	112.89±44.91	113.09±38.18	-0.104	0.917
LDL Cholesterol (mg/dl)	113.49±35.56	111.15±31.42	1.489	0.137	123.2±36.32	125.00±37.35	-1.041	0.298
HDL Cholesterol (mg/dl)	50.53±15.8	52.53±16.25	2.633	0.009	51.62±14.61	52.5±15.33	-1.252	0.211
eGFR(ml/min/1.73m^2^)	81.50±14.52	69.24±15.88	16.964	<0.001	82.21±14.6	69.97±15.68	17.211	<0.001
Serum uric acid(mg/dl)	4.97±1.28	5.14±1.35	-2.682	0.007	4.08±1.13	4.26±1.19	-3.350	0.001
Systolic blood pressure(mmHg)	132±21.28	136.32±26.41	-3.748	<0.001	134.43±25.74	143.46±29.58	-6.885	<0.001
Diastolic blood pressure(mmHg)	77.38±12.52	74.33±12.96	5.036	<0.001	76.83±12.3	75.95±11.98	1.534	0.252
Body mass index(kg/m^2^)	23.06±3.61	21.88±3.70	6.853	<0.001	23.78±3.86	22.90±4.51	4.428	<0.001
Triglycerides (mg/dl)	121.17±99.74	106.04±67.08	3.843	<0.001	142.91±90.84	137.51±97.13	1.223	0.222

**Table 6:** Age-adjusted relationship between various characteristics and blood pressure status of participants categorized by BMI in male (N=1810).

**Variables**	BMI<24(n=1296)		BMI≥24 (n=514)	
	Systolic blood pressure partial r(*P*-value)	Diastolic blood pressure partial r(*P*-value)	Systolic blood pressure partial r(*P*-value)	Diastolic blood pressure partial r(*P*-value)
Education(0= Illiterate, 1= Less than elementary school, 2= High school, 3= Above vocational school)	-0.024(0.392)	0.013(0.647)	-0.009(0.848)	-0.042(0.344)
Marital status(0= Single,1= Married)	-0.105(<0.001)	-0.092(0.001)	0.011(0.807)	-0.011(0.798)
Current residence(0= Rural,1= Urban)	0.044(0.117)	0.032(0.249)	0.113(0.011)	0.059(0.187)
Smoke(0= NO,1= Former smoke,2= Current smoke)	0.06(0.033)	0.009(0.752)	0.066(0.140)	0.016(0.727)
Drinking(0= NO,1= Less than once a month,2= More than once a month)	0.015(0.602)	0.020(0.480)	-0.066(0.139)	-0.003(0.944)
Eating meals(0= ≤2 meals per day,1=3 meals per day,2= ≥4 meals per day)	-0.061(0.031)	-0.083(0.003)	-0.014(0.759)	-0.056(0.21)
Taking activities(0= No,1= Yes)	0.014(0.623)	0.031(0.268)	0.091(0.040)	0.118(0.008)
Ever been in major accidental injury(0= No,1= Yes)	-0.028(0.320)	-0.019(0.505)	-0.035(0.429)	-0.007(0.871)
Having regular physical exercises(0= No physical exercise,1= Less than regular physical exercises,2= Regular physical exercises)	0.034(0.23)	0.006(0.833)	0.011(0.803)	0.011(0.814)
History of CVD(0= No,1= Yes)	0.019(0.497)	0.028(0.327)	-0.009(0.833)	0.008(0.854)
History of liver diseases(0= No,1= Yes)	0.009(0.760)	0.013(0.631)	-0.023(0.613)	0.028(0.526)
Antilipidemic medication(0= No,1= Yes)	-0.008(0.787)	0.019(0.504)	0.002(0.973)	0.027(0.546)
Anti-diabetic medication(0= No,1= Yes)	0.002(0.955)	0.007(0.806)	0.043(0.339)	-0.012(0.796)
Anti-hypertensive therapy(0= No,1= Yes)	0.140(<0.001)	0.109(<0.001)	0.047(0.291)	0.127(0.004)
Fasting plasma glucose(mg/dl)	0.075(0.007)	0.046(0.098)	0.068(0.126)	0.065(0.147)
LDL Cholesterol (mg/dl)	0.063(0.024)	0.052(0.067)	0.063(0.157)	0.024(0.585)
HDL Cholesterol (mg/dl)	-0.005(0.848)	0.005(0.870)	-0.042(0.344)	-0.003(0.942)
eGFR(ml/min/1.73m^2^)	-0.076(0.007)	-0.043(0.124)	-0.157(<0.001)	-0.107(0.016)
Serum uric acid(mg/dl)	0.108(<0.001)	0.06(0.032)	0.056(0.212)	0.067(0.130)
Body mass index(kg/m^2^)	0.121(<0.001)	0.093(0.001)	0.062(0.163)	0.048(0.279)
Triglycerides (mg/dl)	0.069(0.014)	0.058(0.04)	0.074(0.097)	0.047(0.288)

**Table 7:** Age-adjusted relationship between various characteristics and blood pressure status of participants categorized by BMI in female (N=1819).

**Variables**	BMI<24 **(n=1080)**		BMI≥24 **(n=739)**	
	Systolic blood pressure partial r(*P*-value)	Diastolic blood pressure partial r(*P*-value)	Systolic blood pressure partial r(*P*-value)	Diastolic blood pressure partial r(*P*-value)
Education(0= Illiterate, 1= Less than elementary school, 2= High school, 3= Above vocational school)	-0.053(0.086)	-0.07(0.024)	-0.002(0.957)	-0.041(0.270)
Marital status(0= Single,1= Married)	-0.097(0.002)	-0.077(0.012)	-0.066(0.076)	-0.015(0.687)
Current residence(0= Rural,1= Urban)	-0.01(0.745)	-0.039(0.211)	0.039(0.294)	0.035(0.344)
Smoke(0= NO,1= Former smoke,2= Current smoke)	0.035(0.255)	0.021(0.503)	-0.025(0.508)	0.007(0.844)
Drinking(0= NO,1= Less than once a month,2= More than once a month)	-0.016(0.608)	0.015(0.630)	-0.022(0.549)	-0.02(0.584)
Eating meals(0= ≤2 meals per day,1=3 meals per day,2= ≥4 meals per day)	-0.021(0.505)	-0.075(0.015)	-0.026(0.483)	-0.069(0.064)
Taking activities(0= No,1= Yes)	-0.036(0.251)	-0.034(0.266)	0.027(0.474)	0.027(0.472)
Ever been in major accidental injury(0= No,1= Yes)	-0.01(0.745)	0.014(0.640)	-0.041(0.274)	-0.042(0.259)
Having regular physical exercises(0= No physical exercise,1= Less than regular physical exercises,2= Regular physical exercises)	-0.008(0.799)	-0.008(0.803)	-0.015(0.692)	0.031(0.401)
History of CVD(0= No,1= Yes)	0.036(0.239)	0.001(0.986)	-0.014(0.700)	-0.021(0.582)
History of liver diseases(0= No,1= Yes)	0.005(0.868)	0.003(0.934)	-0.047(0.213)	-0.103(0.006)
Antilipidemic medication(0= No,1= Yes)	0.065(0.034)	0.054(0.082)	0.054(0.151)	0.032(0.399)
Anti-diabetic medication(0= No,1= Yes)	0.013(0.686)	-0.029(0.356)	0.122(0.001)	0.046(0.214)
Anti-hypertensive therapy(0= No,1= Yes)	0.110(<0.001)	0.037(0.029)	0.128(<0.001)	0.102(<0.001)
Fasting plasma glucose(mg/dl)	0.049(0.115)	0.043(0.161)	0.021(0.577)	0.158(<0.001)
LDL Cholesterol (mg/dl)	0.028(0.362)	0.011(0.725)	0.049(0.189)	-0.005(0.886)
HDL Cholesterol (mg/dl)	-0.087(0.005)	-0.06(0.051)	-0.039(0.293)	-0.050(0.184)
eGFR(ml/min/1.73m^2^)	-0.069(0.025)	-0.045(0.147)	0.067(0.075)	0.020(0.600)
Serum uric acid(mg/dl)	0.126(<0.001)	0.074(0.016)	-0.018(0.627)	0.011(0.764)
Body mass index(kg/m^2^)	0.105(0.001)	0.093(0.002)	0.021(0.583)	0.075(0.045)
Triglycerides (mg/dl)	0.123(<0.001)	0.091(0.003)	0.046(0.218)	0.097(0.010)

**Table 8:** Relationship between various characteristics and blood pressure status of participants categorized by age in male (N=1810).

**Variables**	Age <68 years (n=807)		Age≥68 years (n=1003)	
	Systolic blood pressure partial r(*P*-value)	Diastolic blood pressure partial r(*P*-value)	Systolic blood pressure partial r(*P*-value)	Diastolic blood pressure partial r(*P*-value)
Age	0.052(0.146)	-0.011(0.763)	0.05(0.116)	-0.096(0.002)
Education(0= Illiterate, 1= Less than elementary school, 2= High school, 3= Above vocational school)	0.013(0.720)	-0.016(0.646)	-0.019(0.544)	0.043(0.173)
Marital status(0= Single,1= Married)	-0.064(0.070)	-0.086(0.015)	-0.074(0.020)	-0.033(0.295)
Current residence(0= Rural,1= Urban)	0.072(0.042)	0.039(0.274)	0.102(0.001)	0.096(0.003)
Smoke(0= NO,1= Former smoke,2= Current smoke)	0.018(0.616)	0.017(0.633)	0.042(0.184)	-0.023(0.462)
Drinking(0= NO,1= Less than once a month,2= More than once a month)	-0.028(0.425)	-0.015(0.679)	-0.006(0.856)	0.037(0.244)
Eating meals(0= ≤2 meals per day,1=3 meals per day,2= ≥4 meals per day)	-0.065(0.067)	-0.098(0.006)	-0.026(0.420)	-0.050(0.114)
Taking activities(0= No,1= Yes)	-0.036(0.315)	-0.040(0.255)	0.022(0.495)	0.042(0.189)
Ever been in major accidental injury(0= No,1= Yes)	-0.044(0.212)	-0.053(0.137)	-0.026(0.416)	-0.001(0.975)
Having regular physical exercises(0= No physical exercise,1= Less than regular physical exercises,2= Regular physical exercises)	-0.001(0.987)	-0.005(0.89)	0.046(0.147)	0.022(0.489)
History of CVD(0= No,1= Yes)	0.055(0.123)	0.06(0.089)	0.009(0.786)	0.027(0.396)
History of liver diseases(0= No,1= Yes)	-0.033(0.357)	-0.019(0.598)	0.024(0.448)	0.056(0.078)
Antilipidemic medication(0= No,1= Yes)	0.060(0.092)	0.06(0.088)	-0.018(0.561)	0.021(0.502)
Anti-diabetic medication(0= No,1= Yes)	0.038(0.281)	-0.002(0.945)	0.041(0.198)	0.045(0.157)
Anti-hypertensive therapy(0= No,1= Yes)	0.131(<0.001)	0.122(0.001)	0.103(0.001)	0.118(<0.001)
Fasting plasma glucose(mg/dl)	0.122(0.001)	0.092(0.009)	0.069(0.031)	0.048(0.131)
LDL Cholesterol (mg/dl)	0.096(0.007)	0.067(0.056)	0.061(0.054)	0.054(0.087)
HDL Cholesterol (mg/dl)	-0.102(0.004)	-0.077(0.028)	-0.049(0.122)	-0.026(0.405)
eGFR(ml/min/1.73m^2^)	-0.119(0.001)	-0.084(0.017)	-0.117(<0.001)	-0.016(0.618)
Serum uric acid(mg/dl)	0.143(<0.001)	0.122(0.001)	0.104(0.001)	0.049(0.123)
Body mass index(kg/m^2^)	0.252(<0.001)	0.218(<0.001)	0.146(<0.001)	0.140(<0.001)
Triglycerides (mg/dl)	0.091(0.010)	0.091(0.010)	0.105(0.001)	0.083(0.009)

**Table 9:** Relationship between various characteristics and blood pressure status of participants categorized by age in female (N=1819).

**Variables**	Age <68 years (n=887)		Age≥68 years (n=932)	
	Systolic blood pressure partial r(*P*-value)	Diastolic blood pressure partial r(*P*-value)	Systolic blood pressure partial r(*P*-value)	Diastolic blood pressure partial r(*P*-value)
Age	0.067(0.048)	-0.032(0.348)	0.117(<0.001)	-0.039(0.244)
Education(0= Illiterate, 1= Less than elementary school, 2= High school, 3= Above vocational school)	-0.033(0.335)	-0.020(0.546)	-0.034(0.308)	-0.051(0.124)
Marital status(0= Single,1= Married)	-0.073(0.031)	-0.028(0.409)	-0.104(0.002)	-0.035(0.292)
Current residence(0= Rural,1= Urban)	0.034(0.320)	0.028(0.410)	0.021(0.536)	-0.007(0.822)
Smoke(0= NO,1= Former smoke,2= Current smoke)	0.043(0.203)	0.024(0.481)	-0.040(0.230)	-0.024(0.471)
Drinking(0= NO,1= Less than once a month,2= More than once a month)	-0.017(0.605)	-0.008(0.818)	-0.028(0.391)	0.001(0.970)
Eating meals(0= ≤2 meals per day,1=3 meals per day,2= ≥4 meals per day)	-0.053(0.118)	-0.07(0.039)	0.013(0.700)	-0.041(0.221)
Taking activities(0= No,1= Yes)	0.022(0.509)	0.053(0.115)	-0.002(0.956)	-0.049(0.142)
Ever been in major accidental injury(0= No,1= Yes)	-0.018(0.589)	0.012(0.719)	-0.034(0.300)	-0.032(0.330)
Having regular physical exercises(0= No physical exercise,1= Less than regular physical exercises,2= Regular physical exercises)	-0.016(0.638)	0.025(0.460)	-0.011(0.735)	0.007(0.834)
History of CVD(0= No,1= Yes)	0.079(0.020)	0.074(0.028)	-0.032(0.332)	-0.057(0.084)
History of liver diseases(0= No,1= Yes)	0.019(0.574)	0.011(0.746)	-0.054(0.104)	-0.088(0.008)
Antilipidemic medication(0= No,1= Yes)	0.095(0.005)	0.075(0.027)	0.058(0.081)	0.046(0.161)
Anti-diabetic medication(0= No,1= Yes)	0.125(<0.001)	0.061(0.069)	0.057(0.087)	-0.004(0.896)
Anti-hypertensive therapy(0= No,1= Yes)	0.096(0.004)	0.060(0.073)	0.135(<0.001)	0.076(0.022)
Fasting plasma glucose(mg/dl)	0.061(0.072)	0.159(<0.001)	0.031(0.344)	0.055(0.097)
LDL Cholesterol (mg/dl)	0.048(0.157)	0.028(0.404)	0.05(0.132)	0.014(0.668)
HDL Cholesterol (mg/dl)	-0.137(<0.001)	-0.118(<0.001)	-0.063(0.058)	-0.085(0.010)
eGFR(ml/min/1.73m^2^)	-0.039(0.248)	-0.007(0.836)	-0.025(0.455)	-0.011(0.743)
Serum uric acid(mg/dl)	0.084(0.012)	0.083(0.013)	0.092(0.005)	0.054(0.100)
Body mass index(kg/m^2^)	0.161(<0.001)	0.253(<0.001)	0.116(<0.001)	0.116(<0.001)
Triglycerides (mg/dl)	0.155(<0.001)	0.148(<0.001)	0.069(0.037)	0.096(0.004)

**Table 10:** Multivariate-adjusted relationship between various characteristics and blood pressure status of participants categorized by BMI in male (N=1810).

**Variables**	BMI<24(n=1296)		BMI≥24 (n=514)	
	Systolic blood pressure *β*(*P*-value)	Diastolic blood pressure *β* (*P*-value)	Systolic blood pressure *β* (*P*-value)	Diastolic blood pressure *β* (*P*-value)
**Age (years)**	0.117(<0.001)	-0.108(0.001)	—	-0.199(<0.001)
Education (0= Illiterate, 1= Less than elementary school, 2= High school, 3= Above vocational school)	—	—	—	—
**Marital status (0= Single,1= Married)**	-0.101(<0.001)	-0.092(0.001)	—	—
**Current residence (0= Rural,1= Urban)**	—	—	0.119(0.009)	—
Smoke (0= NO,1= Former smoke,2= Current smoke)	0.060(0.033)	—	0.104(0.023)	—
Drinking (0= NO,1= Less than once a month,2= More than once a month)	—	—	—	—
Eating meals (0= ≤2 meals per day,1=3 meals per day,2= ≥4 meals per day)	-0.056(0.041)	-0.085(0.002)	—	—
Taking activities (0= No,1= Yes)	—	—	-0.108(0.016)	-0.116(0.01)
Ever been in major accidental injury (0= No,1= Yes)	—	—	—	—
Having regular physical exercises (0= No physical exercise,1= Less than regular physical exercises,2= Regular physical exercises)	—	—	—	—
History of CVD (0= No,1= Yes)	—	—	—	—
History of liver diseases (0= No,1= Yes)	—	—	—	—
Antilipidemic medication (0= No,1= Yes)	—	—	—	—
Anti-diabetic medication (0= No,1= Yes)	—	—	—	—
Anti-hypertensive therapy (0= No,1= Yes)	0.130(<0.001)	0.103(<0.001)	—	0.113(0.011)
Fasting plasma glucose (mg/dl)	0.072(0.016)	—	—	—
LDL Cholesterol (mg/dl)	0.056(0.042)	—	0.091(0.048)	—
HDL Cholesterol (mg/dl)	—	—	—	—
eGFR (ml/min/1.73m^2^)	—	—	-0.172(0.002)	—
Serum uric acid (mg/dl)	0.068(0.029)	—	—	—
Body mass index (kg/m^2^)	0.106(<0.001)	0.076(0.009)	—	—
Triglycerides (mg/dl)	—	—	—	—
R^2^	0.098(<0.001)	0.054(<0.001)	0.087(0.003)	0.089(0.0002)

**Table 11:** Multivariate-adjusted relationship between various characteristics and blood pressure status of participants categorized by BMI in female (N=1819).

**Variables**	BMI<24(n=1080)		BMI≥24 (n=739)	
	Systolic blood pressure *β*(*P*-value)	Diastolic blood pressure *β* (*P*-value)	Systolic blood pressure *β* (*P*-value)	Diastolic blood pressure *β* (*P*-value)
Age (years)	0.168(<0.001)	—	0.191(<0.001)	0.191(<0.001)
Education (0= Illiterate, 1= Less than elementary school, 2= High school, 3= Above vocational school)	—	-0.072(0.030)	—	—
Marital status (0= Single,1= Married)	-0.106(0.001)	-0.084(0.013)	-0.082(0.034)	-0.082(0.034)
Current residence (0= Rural,1= Urban)	—	—	—	—
Smoke (0= NO,1= Former smoke,2= Current smoke)	—	—	—	—
Drinking (0= NO,1= Less than once a month,2= More than once a month)	—	—	—	—
Eating meals (0= ≤2 meals per day,1=3 meals per day,2= ≥4 meals per day)	—	-0.075(0.016)	—	—
Taking activities (0= No,1= Yes)	—	—	—	—
Ever been in major accidental injury (0= No,1= Yes)	—	—	—	—
Having regular physical exercises (0= No physical exercise,1= Less than regular physical exercises,2= Regular physical exercises)	—	—	—	—
History of CVD (0= No,1= Yes)	—	—	—	—
History of liver diseases (0= No,1= Yes)	—	—	—	—
Antilipidemic medication (0= No,1= Yes)	—	—	—	—
Anti-diabetic medication (0= No,1= Yes)	—	—	0.138(0.001)	0.138(0.001)
Anti-hypertensive therapy(0= No,1= Yes)	0.12(<0.001)	—	0.129(0.001)	0.129(0.001)
Fasting plasma glucose(mg/dl)	—	—	—	—
LDL Cholesterol (mg/dl)	—	—	—	—
HDL Cholesterol (mg/dl)	—	—	—	—
eGFR(ml/min/1.73m^2^)	—	—	—	—
Serum uric acid(mg/dl)	0.084(0.016)	—	—	—
Body mass index(kg/m^2^)	0.082(0.009)	0.086(0.008)	—	—
Triglycerides (mg/dl)	0.078(0.025)	—	—	—
R^2^	0.119(<0.001)	0.046(0.001)	0.089(<0.001)	0.072(<0.001)

**Table 12:** Multivariate-adjusted relationship between various characteristics and blood pressure status of participants categorized by age in male (N=1810).

**Variables**	Age <68 years (n=807)		Age≥68 years (n=1003)	
	Systolic blood pressure *β*(*P*-value)	Systolic blood pressure *β*(*P*-value)	Systolic blood pressure *β*(*P*-value)	Systolic blood pressure *β*(*P*-value)
Age	—	—	—	-0.085(0.015)
Education(0= Illiterate, 1= Less than elementary school, 2= High school, 3= Above vocational school)	—	—	—	—
**Marital status(0= Single,1= Married)**	0.078(0.024)	0.1(0.004)	0.074(0.020)	—
**Current residence(0= Rural,1= Urban)**	—	—	0.105(0.002)	0.084(0.014)
Smoke(0= NO,1= Former smoke,2= Current smoke)	—	—	0.078(0.016)	—
Drinking(0= NO,1= Less than once a month,2= More than once a month)	—	—	—	—
Eating meals(0= ≤2 meals per day,1=3 meals per day,2= ≥4 meals per day)	-0.070(0.039)	-0.103(0.003)	—	-0.071(0.025)
Taking activities(0= No,1= Yes)	-0.077(0.027)	—	—	—
Ever been in major accidental injury(0= No,1= Yes)	—	—	—	—
Having regular physical exercises(0= No physical exercise,1= Less than regular physical exercises,2= Regular physical exercises)	—	—	—	—
History of CVD(0= No,1= Yes)	—	—	—	—
History of liver diseases(0= No,1= Yes)	—	—	—	—
Antilipidemic medication(0= No,1= Yes)	—	—	—	—
Anti-diabetic medication(0= No,1= Yes)	—	-0.078(0.046)	—	—
Anti-hypertensive therapy(0= No,1= Yes)	0.09(0.009)	0.090(0.010)	0.111(<0.001)	0.114(<0.001)
Fasting plasma glucose(mg/dl)	0.09(0.022)	—	—	—
LDL Cholesterol (mg/dl)	—	—	—	—
HDL Cholesterol (mg/dl)	—	—	—	—
eGFR(ml/min/1.73m^2^)	-0.078(0.040)	—	—	—
Serum uric acid(mg/dl)	0.088(0.024)	0.08(0.042)	—	—
Body mass index(kg/m^2^)	0.229(<0.001)	0.213(<0.001)	0.137(<0.001)	0.105(0.003)
Triglycerides (mg/dl)	0.024(0.545)	0.03(0.452)	0.096(0.009)	0.063(0.088)
R^2^	0.136(<0.001)	0.115(<0.001)	0.087(<0.001)	0.065(<0.001)

**Table 13:** Multivariate-adjusted relationship between various characteristics and blood pressure status of participants categorized by age in female (N=1819).

**Variables**	Age <68 years (n=887)		Age≥68 years (n=932)	
	Systolic blood pressure *β*(*P*-value)	Systolic blood pressure *β*(*P*-value)	Systolic blood pressure *β*(*P*-value)	Systolic blood pressure *β*(*P*-value)
Age	—	—	0.105(0.005)	—
Education (0= Illiterate, 1= Less than elementary school, 2= High school, 3= Above vocational school)	—	—	—	—
**Marital status (0= Single,1= Married)**	0.071(0.035)	—	0.103(0.003)	0.071(0.043)
**Current residence (0= Rural,1= Urban)**	—	—	—	—
Smoke (0= NO,1= Former smoke,2= Current smoke)	—	—	—	—
Drinking (0= NO,1= Less than once a month,2= More than once a month)	—	—	—	—
Eating meals (0= ≤2 meals per day,1=3 meals per day,2= ≥4 meals per day)	—	-0.08(0.015)	—	—
Taking activities (0= No,1= Yes)	—	—	—	—
Ever been in major accidental injury (0= No,1= Yes)	—	—	—	—
Having regular physical exercises (0= No physical exercise,1= Less than regular physical exercises,2= Regular physical exercises)	—	—	—	—
History of CVD (0= No,1= Yes)	—	—	—	-0.069(0.048)
History of liver diseases (0= No,1= Yes)	—	—	—	-0.076(0.023)
Antilipidemic medication (0= No,1= Yes)	—	—	—	—
Anti-diabetic medication (0= No,1= Yes)	0.105(0.004)	—	—	—
Anti-hypertensive therapy (0= No,1= Yes)	0.085(0.012)	—	0.153(<0.001)	0.089(0.008)
Fasting plasma glucose (mg/dl)	—	0.128(<0.001)	—	—
LDL Cholesterol (mg/dl)	—	—	—	—
HDL Cholesterol (mg/dl)	—	—	—	—
eGFR (ml/min/1.73m^2^)	—	—	—	—
Serum uric acid (mg/dl)	—	—	—	—
Body mass index (kg/m^2^)	0.11(0.003)	0.23(<0.001)	0.097(0.007)	0.105(0.004)
Triglycerides (mg/dl)	0.087(0.031)	0.056(0.159)	0.046(0.270)	0.072(0.090)
R^2^	0.087(<0.001)	0.110(<0.001)	0.079(<0.001)	0.057(<0.001)

**Table 14:** Interaction between body mass index and uric acid on blood pressure status in male and female (N=3629).

Characteristics	Male(n=1810)		Female(n=1819)	
	Systolic blood pressure *β*(*P*-value)	Diastolic blood pressure *β*(*P*-value)	Systolic blood pressure *β*(*P*-value)	Diastolic blood pressure *β*(*P*-value)
Age(years)	0.337(0.001)	-0.240(<0.001)	-0.092(0.042)	-0.113(0.013)
Education(0= Illiterate, 1= Less than elementary school, 2= High school, 3= Above vocational school)	**—**	**—**	**—**	-1.119(0.019)
Marital status(0= Single,1= Married)	-5.660(0.001)	-2.494(0.004)	-1.503(0.024)	-1.453(0.029)
Current residence(0= Rural,1= Urban)	3.574(0.003)	**—**	**—**	**—**
Smoke(0= NO,1= Former smoke,2= Current smoke)	1.864(0.005)	**—**	**—**	**—**
Eating meals(0= ≤2 meals per day,1=3 meals per day,2= ≥4 meals per day)	2.978(0.045)	2.483(0.002)	**—**	2.193(0.003)
Taking activities(0= No,1= Yes)	**—**	**—**	**—**	**—**
Anti-diabetic medication(0= No,1= Yes)	**—**	**—**	**—**	**—**
Anti-hypertensive therapy(0= No,1= Yes)	10.228(<0.001)	5.745(<0.001)	3.292(0.003)	**—**
Fasting plasma glucose(mg/dl)	0.039(0.011)	**—**	**—**	**—**
LDL Cholesterol (mg/dl)	0.049(0.004)	**—**	**—**	**—**
eGFR(ml/min/1.73m^2^)	-0.123(0.003)	**—**	**—**	**—**
Serum uric acid(mg/dl)	0.948(0.047)	**—**	**—**	**—**
Body mass index	-8.004(0.002)	-4.125(0.002)	-3.668(<0.001)	-4.102(<0.001)
Triglycerides	-3.962(0.076)	-1.482(0.203)	-2.140(0.017)	-2.319(0.009)
Body mass index*∗* Triglycerides	0.572(0.845)	-0.373(0.810)	0.122(0.923)	0.272(0.828)

**Table 15:** Interaction between body mass index and uric acid on blood pressure status of participants categorized by age in male and female (N=3629).

Characteristics	Age <68 years (n=807)		Age≥68 years (n=1003)	

**Male**	Systolic blood pressure *β*(*P*-value)	Diastolic blood pressure *β*(*P*-value)	Systolic blood pressure *β*(*P*-value)	Diastolic blood pressure *β*(*P*-value)

Marital status (0= Single,1= Married)	-6.913(0.009)	-5.237(0.001)	-5.876(0.007)	
Current residence (0= Rural,1= Urban)			5.232(0.004)	2.164(0.015)
Smoke (0= NO,1= Former smoke,2= Current smoke)			1.917(0.05)	
Eating meals (0= ≤2 meals per day,1=3 meals per day,2= ≥4 meals per day)	3.746(0.058)	3.227(0.006)		2.239(0.036)
Taking activities (0= No,1= Yes)	-2.747(0.057)			
Antilipidemic medication (0= No,1= Yes)		-1.651(0.423)		
Anti-hypertensive therapy (0= No,1= Yes)	9.032(0.002)	5.343(0.002)	11.982(<0.001)	6.384(<0.001)
Fasting plasma glucose (mg/dl)	0.040(0.030)			
eGFR (ml/min/1.73m^2^)	-0.122(0.024)			
Serum uric acid(mg/dl)	1.454(0.022)	0.957(0.006)		
Body mass index	-8.409(0.006)	-4.837(0.008)	-8.347(0.048)	-3.042(0.142)
Triglycerides	-0.135(0.958)	-0.587(0.695)	-7.960(0.035)	-1.478(0.427)
Body mass index*∗* Triglycerides	-1.075(0.760)	-0.095(0.964)	2.138(0.654)	-0.931(0.691)

**Female**	Age <68 years (n=887)		Age≥68 years (n=932)	
	Systolic blood pressure *β*(*P*-value)	Diastolic blood pressure *β*(*P*-value)	Systolic blood pressure *β*(*P*-value)	Diastolic blood pressure *β*(*P*-value)

Age	—	—	0.626(0.001)	—
Education (0= Illiterate, 1= Less than elementary school, 2= High school, 3= Above vocational school)	—	—	—	—
Marital status (0= Single,1= Married)	5.866(0.012)	—	5.481(0.007)	7.504(<0.001)
Eating meals (0= ≤2 meals per day,1=3 meals per day,2= ≥4 meals per day)	—	2.821(0.009)	—	—
History of CVD (0= No,1= Yes)	—	—	—	-3.837(0.123)
Antilipidemic medication (0= No,1= Yes)	—	—	—	—
Anti-diabetic medication (0= No,1= Yes)	11.822(0.002)	—	—	—
Anti-hypertensive therapy (0= No,1= Yes)	8.946(0.006)	—	17.459(<0.001)	17.254(<0.001)
Fasting plasma glucose (mg/dl)	—	0.035(<0.001)	—	
Body mass index	-5.696(0.062)	-6.621(<0.001)	-4.726(0.187)	-5.152(0.155)
Triglycerides	-4.716(0.063)	-2.716(0.024)	-1.573(0.622)	-2.147(0.507)
Body mass index*∗* Triglycerides	-1.345(0.718)	2.410(0.170)	-4.192(0.334)	-3.386(0.439)

**Table 16:** Adjusting ORs and 95%CI for BMI or TG and hypertension in male and female.

	Male						Female					
	BMI and hypertension			TG and hypertension			BMI and hypertension			TG and hypertension		
	*OR*	*95*%*CI*	*P*	*OR*	*95*%*CI*	*P*	*OR*	*95*%*CI*	*P*	*OR*	*95*%*CI*	*P*
Age(years)	1.027	(1.007,1.047)	0.007	1.021	(1.002,1.041)	0.029	1.056	(1.037,1.076)	<0.001	1.054	(1.035,1.073)	<0.001
education												
Illiterate	1.000			1.000			1.000			1.000		
Less than elementary school	1.066	(0.817,1.391)	0.636	1.086	(0.834,1.415)	0.538	0.875	(0.703,1.088)	0.229	0.876	(0.705,1.089)	0.233
High school	0.649	(0.271,1.553)	0.331	0.662	(0.279,1.567)	0.348	0.169	(0.037,0.777)	0.022	0.178	(0.038,0.823)	0.027
Above vocational school	0.817	(0.493,1.352)	0.431	0.837	(0.508,1.379)	0.485	0.671	(0.304,1.484)	0.325	0.704	(0.319,1.557)	0.387
Marital status												
Single	1.000			1.000			1.000			1.000		
Married	0.693	(0.515,0.933)	0.016	0.726	(0.540,0.975)	0.033	0.833	(0.660,1.052)	0.126	0.854	(0.676,1.077)	0.183
Current residence												
Urban	1.000			1.000			1.000			1.000		
Rural	1.281	(1.021,1.608)	0.033	1.327	(1.058,1.663)	0.014	1.028	(0.826,1.278)	0.807	1.057	(0.851,1.314)	0.616
Smoke												
NO smoke	1.000			1.000			1.000			1.000		
Former smoke	0.892	(0.660,1.204)	0.454	0.906	(0.672,1.221)	0.518	0.914	(0.523,1.599)	0.753	0.844	(0.482,1.476)	0.551
Current smoke	1.309	(1.019,1.682)	0.035	1.235	(0.964,1.582)	0.095	1.155	(0.810,1.648)	0.427	1.106	(0.777,1.573)	0.577
Drinking												
NO	1.000			1.000			1.000			1.000		
Less than once a month	1.030	(0.823,1.289)	0.796	1.051	(0.841,1.313)	0.663	1.060	(0.733,1.533)	0.758	1.045	(0.722,1.513)	0.814
More than once a month	1.134	(0.772,1.665)	0.523	1.141	(0.780,1.671)	0.496	0.696	(0.416,1.164)	0.167	0.716	(0.429,1.196)	0.202
Eating meals												
≤2 meals per day	1.000			1.000			1.000			1.000		
3 meals per day	0.898	(0.381,2.117)	0.806	0.832	(0.355,1.953)	0.673	0.777	(0.328,1.837)	0.565	0.782	(0.330,1.851)	0.575
≥4 meals per day	0.658	(0.494,0.875)	0.004	0.682	(0.513,0.906)	0.008	0.983	(0.739,1.308)	0.909	1.050	(0.790,1.396)	0.737
Taking no activities												
No	1.000			1.000			1.000			1.000		
Yes	0.916	(0.744,1.127)	0.407	0.930	(0.756,1.142)	0.487	1.021	(0.834,1.250)	0.840	1.035	(0.846,1.266)	0.738
Ever been in major accidental injury												
NO	1.000			1.000			1.000			1.000		
Yes	0.746	(0.535,1.041)	0.085	0.754	(0.542,1.049)	0.094	0.864	(0.584,1.279)	0.4660	0.871	(0.588,1.290)	0.491
Having regular physical exercises												
No physical exercise	1.000			1.000			1.000			1.000		
Less than regular physical exercises	1.199	(0.927,1.550)	0.168	1.220	(0.945,1.576)	0.127	0.975	(0.748,1.270)	0.849	0.959	(0.736,1.250)	0.756
Regular physical exercises	1.282	(0.972,1.690)	0.079	1.292	(0.981,1.701)	0.068	0.919	(0.705,1.197)	0.530	0.922	(0.708,1.201)	0.546
History of CVD												
NO	1.000			1.000			1.000			1.000		
Yes	1.170	(0.869,1.575)	0.300	1.247	(0.930,1.673)	0.141	0.982	(0.750,1.286)	0.896	0.986	(0.753,1.290)	0.916
History of liver diseases												
NO	1.000			1.000			1.000			1.000		
Yes	0.640	(0.369,1.110)	0.112	0.654	(0.379,1.129)	0.128	0.795	(0.466,1.355)	0.399	0.837	(0.491,1.429)	0.515
Antilipidemic medication												
NO	1.000			1.000			1.000			1.000		
Yes	0.984	(0.601,1.612)	0.949	1.027	(0.629,1.679)	0.914	1.711	(1.127,2.596)	0.012	1.783	(1.177,2.701)	0.006
Anti-diabetic medication												
NO	1.000			1.000			1.000			1.000		
Yes	0.549	(0.312,0.965)	0.037	0.586	(0.334,1.030)	0.063	1.421	(0.846,2.387)	0.184	1.540	(0.919,2.581)	0.101
Anti-hypertensive therapy												
NO	1.000			1.000			1.000			1.000		
Yes	2.448	(1.635,3.665)	<0.001	2.522	(1.690,3.766)	<0.001	2.097	(1.416,3.105)	<0.001	2.154	(1.457,3.186)	<0.001
Fasting plasma glucose(mg/dl)	1.005	(1.002,1.008)	<0.001	1.005	(1.002,1.008)	0.001	1.004	(1.001,1.007)	0.005	1.004	(1.001,1.006)	0.017
LDL Cholesterol (mg/dl)	1.004	(1.001,1.007)	0.014	1.004	(1.001,1.008)	0.004	1.001	(0.998,1.004)	0.585	1.001	(0.999,1.004)	0.316
HDL Cholesterol (mg/dl)	0.998	(0.991,1.005)	0.614	0.995	(0.988,1.002)	0.158	0.997	(0.990,1.004)	0.383	0.999	(0.991,1.006)	0.708
Egfr (ml/min/1.73m^2^)	0.993	(0.986,1.001)	0.089	0.993	(0.986,1.001)	0.091	0.998	(0.990,1.005)	0.566	0.997	(0.990,1.005)	0.455
Serum uric acid(mg/dl)	1.160	(1.064,1.265)	0.001	1.175	(1.077,1.282)	<0.001	1.122	(1.017,1.238)	0.022	1.118	(1.013,1.234)	0.027
Body mass index (kg/m^2^)												
BMI <24	1.000						1.000					
BM I≥24	1.781	(1.393,2.277)	<0.001				1.653	(1.330,2.055)	<0.001			
Triglycerides (mg/dl)												
<150				1.000						1.000		
≥150				1.169	(0.882,1.548)	0.277				1.558	(1.219,1.992)	<0.001

**Table 17:** Adjusting ORs and 95%CI for BMI or TG and hypertension categorized by age in male.

	Age <68 years (n=807)						Age≥68 years (n=1003)					
	BMI and hypertension			TG and hypertension			BMI and hypertension			TG and hypertension		
	*OR*	*95*%*CI*	*P*	*OR*	*95*%*CI*	*P*	*OR*	*95*%*CI*	*P*	*OR*	*95*%*CI*	*P*
Age(years)	1.009	(0.937,1.088)	0.806	1.006	(0.934,1.083)	0.877	1.021	(0.988,1.055)	0.212	1.015	(0.982,1.048)	0.383
education												
Illiterate	1.000			1.000			1.000			1.000		
Less than elementary school	0.805	(0.487,1.330)	0.397	0.850	(0.516,1.401)	0.523	1.171	(0.852,1.609)	0.330	1.182	(0.862,1.621)	0.299
High school	1.212	(0.353,4.157)	0.760	1.201	(0.352,4.092)	0.770	0.303	(0.080,1.158)	0.081	0.321	(0.086,1.207)	0.093
Above vocational school	0.687	(0.288,1.641)	0.399	0.749	(0.316,1.774)	0.511	0.809	(0.424,1.543)	0.519	0.802	(0.423,1.521)	0.499
Marital status												
Single	1.000			1.000			1.000			1.000		
Married	0.501	(0.283,0.885)	0.017	0.548	(0.311,0.967)	0.038	0.762	(0.535,1.085)	0.132	0.785	(0.552,1.116)	0.177
Current residence												
Urban	1.000			1.000			1.000			1.000		
Rural	1.183	(0.834,1.680)	0.346	1.244	(0.880,1.759)	0.216	1.413	(1.037,1.925)	0.028	1.459	(1.072,1.986)	0.016
Smoke												
NO smoke	1.000			1.000			1.000			1.000		
Former smoke	0.894	(0.538,1.486)	0.666	0.897	(0.541,1.486)	0.672	0.905	(0.617,1.327)	0.609	0.927	(0.634,1.356)	0.696
Current smoke	1.337	(0.893,2.000)	0.158	1.219	(0.820,1.810)	0.327	1.322	(0.953,1.836)	0.095	1.274	(0.921,1.763)	0.144
Drinking												
NO	1.000			1.000			1.000			1.000		
Less than once a month	0.971	(0.681,1.386)	0.873	0.996	(0.699,1.418)	0.980	1.049	(0.778,1.413)	0.755	1.064	(0.791,1.431)	0.681
More than once a month	.908	(0.504,1.636)	0.747	0.918	(0.512,1.645)	0.774	1.459	(0.863,2.469)	0.159	1.469	(0.872,2.475)	0.148
Eating meals												
≤2 meals per day	1.000			1.000			1.000			1.000		
3 meals per day	0.093	(0.011,0.803)	0.031	0.090	(0.011,0.756)	0.027	3.274	(0.921,11.630)	0.067	3.131	(0.884,11.09)	0.077
≥4 meals per day	0.624	(0.396,0.983)	0.042	0.644	(0.408,1.016)	0.058	0.657	(0.452,0.955)	0.028	0.684	(0.472,0.992)	0.045
Taking no activities												
No	1.000			1.000			1.000			1.000		
Yes	0.748	(0.541,1.035)	0.080	0.764	(0.554,1.054)	0.101	1.053	(0.798,1.389)	0.717	1.065	(0.808,1.403)	0.657
Ever been in major accidental injury												
NO	1.000			1.000			1.000			1.000		
Yes	0.640	(0.363,1.128)	0.122	0.606	(0.344,1.065)	0.082	0.781	(0.510,1.197)	0.256	0.809	(0.530,1.234)	0.325
Having regular physical exercises												
No physical exercise	1.000			1.000			1.000			1.000		
Less than regular physical exercises	0.959	(0.626,1.468)	0.846	0.978	(0.641,1.494)	0.919	1.397	(1.000,1.952)	0.050	1.418	(1.017,1.978)	0.039
Regular physical exercises	1.185	(0.776,1.809)	0.432	1.225	(0.805,1.864)	0.344	1.432	(0.983,2.084)	0.061	1.422	(0.978,2.066)	0.065
History of CVD												
NO	1.000			1.000			1.000			1.000		
Yes	1.355	(0.821,2.238)	0.235	1.478	(0.902,2.424)	0.121	1.063	(0.725,1.557)	0.755	1.132	(0.777,1.650)	0.518
History of liver diseases												
NO	1.000			1.000			1.000			1.000		
Yes	0.630	(0.283,1.399)	0.256	.624	(0.283,1.378)	0.244	0.595	(0.270,1.309)	0.197	0.640	(0.293,1.395)	0.261
Antilipidemic medication												
NO	1.000			1.000			1.000			1.000		
Yes	1.135	(0.568,2.269)	0.720	1.142	(0.574,2.273)	0.706	0.861	(0.407,1.821)	0.695	0.911	(0.433,1.902)	0.807
Anti-diabetic medication												
NO	1.000			1.000			1.000			1.000		
Yes	0.476	(0.194,1.173)	0.107	0.520	(0.210,1.285)	0.156	0.655	(0.308,1.393)	0.272	0.683	(0.323,1.447)	0.320
Anti-hypertensive therapy												
NO	1.000			1.000			1.000			1.000		
Yes	2.277	(1.242,4.174)	0.008	2.299	(1.258,4.202)	0.007	2.681	(1.536,4.681)	0.001	2.748	(1.577,4.791)	<0.001
Fasting plasma glucose(mg/dl)	1.004	(1.000,1.008)	0.075	1.004	(1.000,1.009)	0.071	1.007	(1.002,1.011)	0.003	1.006	(1.002,1.011)	0.005
LDL Cholesterol (mg/dl)	1.007	(1.003,1.011)	0.002	1.008	(1.003,1.012)	0.001	1.001	(0.996,1.005)	0.817	1.001	(0.997,1.005)	0.655
HDL Cholesterol (mg/dl)	0.993	(0.982,1.004)	0.227	0.989	(0.977,1.000)	0.053	1.002	(0.993,1.011)	0.691	0.998	(0.989,1.008)	0.743
eGFR(ml/min/1.73m^2^)	0.987	(0.976,0.999)	0.040	0.988	(0.976,1.000)	0.048	0.998	(0.988,1.008)	0.692	0.998	(0.988,1.008)	0.682
Serum uric acid(mg/dl)	1.171	(1.020,1.344)	0.025	1.184	(1.028,1.364)	0.019	1.185	(1.056,1.331)	0.004	1.205	(1.074,1.353)	0.002
Body mass index(kg/m^2^)												
BMI<24	1.000						1.000					
BMI≥24	1.805	(1.249,2.610)	0.002				1.796	(1.275,2.529)	0.001			
Triglycerides (mg/dl)												
<150				1.000						1.000		
≥150				1.140	(0.754,1.722)	0.535				1.098	(0.733,1.643)	0.651

**Table 18:** Adjusting ORs and 95%CI for BMI or TG and hypertension categorized by age in female.

	Age <68 years (n=807)						Age≥68 years (n=1003)					
	BMI and hypertension			TG and hypertension			BMI and hypertension			TG and hypertension		
	*OR*	*95*%*CI*	*P*	*OR*	*95*%*CI*	*P*	*OR*	*95*%*CI*	*P*	*OR*	*95*%*CI*	*P*
Age(years)	1.077	(1.008,1.105)	0.029	1.071	(1.003,1.144)	0.040	1.055	(1.022,1.089)	0.001	1.053	(1.020,1.087)	0.001
education												
Illiterate	1.000			1.000			1.000			1.000		
Less than elementary school	0.807	(0.596,1.093)	0.166	0.803	(0.593,1.087)	0.155	0.966	(0.698,1.337)	0.834	0.972	(0.702,1.345)	0.863
High school	0.231	(0.027,1.980)	0.181	0.277	(0.032,2.410)	0.245	0.121	(0.014,1.046)	0.055	0.116	(0.013,1.003)	0.050
Above vocational school	0.798	(0.227,2.805)	0.725	0.820	(0.239,2.809)	0.752	0.668	(0.234,1.907)	0.451	0.726	(0.252,2.086)	0.552
Marital status												
Single	1.000			1.000			1.000			1.000		
Married	0.681	(0.457,1.017)	0.060	0.717	(0.482,1.066)	0.100	0.891	(0.663,1.196)	0.441	0.904	(0.674,1.214)	0.502
Current residence												
Urban	1.000			1.000			1.000			1.000		
Rural	1.118	(0.810,1.543)	0.499	1.191	(0.864,1.643)	0.286	1.003	(0.739,1.362)	0.984	1.009	(0.743,1.369)	0.955
Smoke												
NO smoke	1.000			1.000			1.000			1.000		
Former smoke	2.322	(0.771,6.998)	0.134	2.228	(0.743,6.681)	0.153	0.715	(0.367,1.391)	0.323	0.649	(0.333,1.267)	0.206
Current smoke	1.502	(0.905,2.494)	0.116	1.383	(0.840,2.277)	0.203	0.888	(0.535,1.473)	0.644	0.858	(0.517,1.424)	0.553
Drinking												
NO	1.000			1.000			1.000			1.000		
Less than once a month	0.917	(0.518,1.622)	0.765	0.963	(0.547,1.695)	0.896	1.107	(0.668,1.837)	0.693	1.057	(0.635,1.758)	0.831
More than once a month	0.938	(0.464,1.897)	0.859	0.894	(0.443,1.803)	0.755	0.508	(0.235,1.100)	0.086	0.559	(0.257,1.214)	0.142
Eating meals												
≤2 meals per day	1.000			1.000			1.000			1.000		
3 meals per day	0.843	(0.264,2.689)	0.773	0.861	(0.269,2.754)	0.800	0.637	(0.176,2.302)	0.492	0.646	(0.179,2.332)	0.505
≥4 meals per day	0.744	(0.486,1.140)	0.174	0.801	(0.524,1.225)	0.306	1.213	(0.822,1.790)	0.330	1.297	(0.879,1.914)	0.190
Taking no activities												
No	1.000			1.000			1.000			1.000		
Yes	0.950	(0.705,1.280)	0.737	0.984	(0.732,1.323)	0.917	1.117	(0.8400,1.486)	0.446	1.115	(0.838,1.482)	0.456
Ever been in major accidental injury												
NO	1.000			1.000			1.000			1.000		
Yes	0.888	(0.491,1.604)	0.693	0.906	(0.501,1.639)	0.744	0.831	(0.485,1.423)	0.499	0.820	(0.477,1.408)	.472
Having regular physical exercises												
No physical exercise	1.000			1.000			1.000			1.000		
Less than regular physical exercises	1.037	(0.709,1.517)	0.852	1.024	(0.701,1.496)	0.904	0.851	(0.581,1.246)	0.407	0.826	(0.564,1.210)	.327
Regular physical exercises	0.978	(0.669,1.432)	0.911	0.953	(0.652,1.393)	0.804	0.924	(0.627,1.361)	0.688	0.944	(0.640,1.391)	.770
History of CVD												
NO	1.000			1.000			1.000			1.000		
Yes	1.563	(1.062,2.300)	0.023	1.586	(1.079,2.331)	0.019	0.640	(0.435,0.942)	0.023	0.640	(0.435,0.940)	0.023
History of liver diseases												
NO	1.000			1.000			1.000			1.000		
Yes	1.100	(0.495,2.446)	0.815	1.190	(0.533,2.654)	0.671	0.645	(0.309,1.345)	0.242	0.671	(0.322,1.398)	0.286
Antilipidemic medication												
NO	1.000			1.000			1.000			1.000		
Yes	1.487	(0.869,2.544)	0.148	1.587	(0.930,2.708)	0.090	2.127	(1.051,4.301)	0.036	2.174	(1.082,4.368)	0.029
Anti-diabetic medication												
NO	1.000			1.000			1.000			1.000		
Yes	1.613	(0.806,3.229)	0.177	1.728	(0.865,3.454)	0.122	1.318	(0.594,2.925)	0.498	1.429	(0.648,3.147)	0.376
Anti-hypertensive therapy												
NO	1.000			1.000			1.000			1.000		
Yes	2.039	(1.187,3.502)	0.010	2.031	(1.185,3.479)	0.010	2.260	(1.241,4.113)	0.008	2.409	(1.321,4.396)	0.004
Fasting plasma glucose(mg/dl)	1.003	(1.000,1.007)	0.077	1.003	(0.999,1.007)	0.108	1.005	(1.001,1.009)	0.027	1.004	(1.000,1.008)	0.081
LDL Cholesterol (mg/dl)	0.999	(0.995,1.003)	0.686	1.000	(0.996,1.004)	0.836	1.002	(0.998,1.006)	0.331	1.002	(0.998,1.006)	0.245
HDL Cholesterol (mg/dl)	0.997	(0.986,1.008)	0.629	0.998	(0.986,1.010)	0.745	0.995	(0.985,1.005)	0.344	0.998	(0.987,1.008)	0.688
eGFR(ml/min/1.73m^2^)	1.001	(0.990,1.012)	0.845	1.001	(0.990,1.012)	0.922	0.996	(0.985,1.006)	0.436	0.995	(0.984,1.005)	0.331
Serum uric acid(mg/dl)	1.132	(0.979,1.310)	0.095	1.117	(0.965,1.294)	0.137	1.103	(0.961,1.265)	0.163	1.099	(0.958,1.262)	0.179
Body mass index(kg/m^2^)												
BMI<24	1.000						1.000					
BMI≥24	1.936	(1.404,2.668)	<0.001				1.506	(1.108,2.047)	0.009			
Triglycerides (mg/dl)												
<150				1.000						1.000		
≥150				1.629	(1.149,2.309)	0.006				1.596	(1.113,2.288)	0.011

## Data Availability

Data sharing statement Extra data can be accessed via http://charls.pku.edu.cn/zh-CN.

## Abbreviations

CHARLS: China Health and Retirement Longitudinal Study
BMI: Body mass index
BP: Blood pressure
DBP: Diastolic blood pressure
SBP: Systolic blood pressure
SUA: Serum uric acid
CVD: Cardiovascular disease
M: Mean
eGFR: Estimated glomerular filtration rate
CDC: Centers for Disease Control and Prevention
Scr: Serum creatinine
Scys: Serum cystatin C
SD: Standard deviation
LDL: Low-density lipoprotein
HDL: High-density lipoprotein
NIA: National intelligence agency
FPG: Fasting plasma glucose
TG: Triglycerides
WC: Waist circumference
WHR: Waist to hip ratio.
